# Region-specific amyloid-β accumulation in the olfactory system influences olfactory sensory neuronal dysfunction in 5xFAD mice

**DOI:** 10.1186/s13195-020-00730-2

**Published:** 2021-01-04

**Authors:** Gowoon Son, Seung-Jun Yoo, Shinwoo Kang, Ameer Rasheed, Da Hae Jung, Hyunjun Park, Bongki Cho, Harry W. M. Steinbusch, Keun-A Chang, Yoo-Hun Suh, Cheil Moon

**Affiliations:** 1 Department of Brain & Cognitive Sciences, Graduate School, Daegu Gyeungbuk Institute of Science and Technology (DGIST), Daegu, Republic of Korea; 2 School for Mental Health and Neuroscience, Maastricht University, Maastricht, the Netherlands; 3 Convergence Research Advanced Centre for Olfaction, Daegu Gyeungbuk Institute of Science and Technology (DGIST), Daegu, Republic of Korea; 4 Max Planck Research Unit for Neurogenetics, Frankfurt, Germany; 5 Department of Pharmacology, College of Medicine, Gachon University, Incheon, Republic of Korea; 6 Korea Brain Research Institute, Daegu, Republic of Korea

**Keywords:** Alzheimer’s disease, Olfactory dysfunction, β-amyloid, 5xFAD, Olfactory sensory neuron, Zonal organization, Odor detection test, Ca^2+^ imaging, Topographic analysis, Neuronal turnover

## Abstract

**Background:**

Hyposmia in Alzheimer’s disease (AD) is a typical early symptom according to numerous previous clinical studies. Although amyloid-β (Aβ), which is one of the toxic factors upregulated early in AD, has been identified in many studies, even in the peripheral areas of the olfactory system, the pathology involving olfactory sensory neurons (OSNs) remains poorly understood.

**Methods:**

Here, we focused on peripheral olfactory sensory neurons (OSNs) and delved deeper into the direct relationship between pathophysiological and behavioral results using odorants. We also confirmed histologically the pathological changes in 3-month-old 5xFAD mouse models, which recapitulates AD pathology. We introduced a numeric scale histologically to compare physiological phenomenon and local tissue lesions regardless of the anatomical plane.

**Results:**

We observed the odorant group that the 5xFAD mice showed reduced responses to odorants. These also did not physiologically activate OSNs that propagate their axons to the ventral olfactory bulb. Interestingly, the amount of accumulated amyloid-β (Aβ) was high in the OSNs located in the olfactory epithelial ectoturbinate and the ventral olfactory bulb glomeruli. We also observed irreversible damage to the ectoturbinate of the olfactory epithelium by measuring the impaired neuronal turnover ratio from the basal cells to the matured OSNs.

**Conclusions:**

Our results showed that partial and asymmetrical accumulation of Aβ coincided with physiologically and structurally damaged areas in the peripheral olfactory system, which evoked hyporeactivity to some odorants. Taken together, partial olfactory dysfunction closely associated with peripheral OSN’s loss could be a leading cause of AD-related hyposmia, a characteristic of early AD.

**Supplementary Information:**

The online version contains supplementary material available at 10.1186/s13195-020-00730-2.

## Background

Olfactory dysfunction affects approximately 90% of patients with Alzheimer’s disease (AD) [1, 2]. Abnormal olfaction has been recognized as one of the earliest clinical manifestations of AD over the past few decades [3, 4]. Numerous attempts have been made to comprehend early AD by using olfaction-based assessments [5–7]. Recent research has shown that patients with AD who have difficulty with olfaction are not completely anosmic [8]. According to the results of clinical studies, patients have difficulty identifying only a few odorants presented, and not all odorants [9, 10]. Besides, the early-onset issues have sparked the mechanism study of hyposmia to understand AD progression. Interestingly, although amyloid-β (Aβ), which is one of the toxic factors upregulated early in AD, has been identified in many studies, even in the peripheral areas of the olfactory system [11–13], the pathology involving olfactory sensory neurons (OSNs) remains poorly understood.

Odor detection via OSNs is the first step in smell processing. The signals converge in the glomerulus in the olfactory bulb (OB), where synapses form between the axon terminal of OSNs and the dendrites of mitral, periglomerular, and tufted cells [14, 15]. Each OSN expresses one type of odorant receptor, and its axon propagates to the OB. The axons of OSNs that express the same odorant receptor form two or three glomeruli per each bulb, which follows a topological map or axis [16, 17]. The OSNs targeted to ventral glomeruli are located in the ectoturbinate of the olfactory epithelium (OE), whereas the OSNs targeted to the dorsal glomeruli are located in the endoturbinate of the OE [18–21]. We also previously demonstrated a distinctive accumulation pattern of Aβ oligomers with specifically β-secretase 1 (BACE1) expression in the OSNs’ terminal located in the OB. This suggested that the uneven partial spatial damage leads to abnormal AD hyposmic symptoms in the olfactory system [11]*.* However, it has not been known how partial damage of the olfactory systems is causally related to distinct olfactory dysfunctions nor has a clear mechanism been proposed that induces damage to the olfactory epithelium by the accumulation of Aβ oligomers. In this study, we delve deeper into the functional impairment related to the spatial distribution of accumulated Aβ oligomers.

We observed a distinctive pattern of hyposmia using an olfactory detection test and electrophysiological changes in OSNs in the early stage of oligomerized Aβ accumulation. We also revealed the spatial correlation between the functional changes and immunohistological signals using a heat map analysis. Given the characterization of the olfactory topographical axis with intrinsic turnover, we identified an interrelation between Aβ oligomer deposits and the breakdown of OSN turnover. To explain the role of Aβ in hyposmia, we used transgenic mice with early-onset familial forms of AD (FAD). These mice have elevated Aβ levels caused by the coexpression of five FAD (5xFAD) mutations (three in amyloid precursor protein [APP] and two in presenilin 1 [PS1]) [22]. This model develops a large Aβ_42_ load (known to generate toxic oligomerized species of Aβs) starting at 2 months of age [22]. It is also well known that these mice develop central nervous system (CNS) deficits after 5 months [22]. Thus, we used 3-month-old 5xFAD mice as a means of identifying a potential causative role for Aβ-derived neurotoxicity in early AD [22]. In particular, we here established a tool for monitoring the pathological features of the OSNs. We confirmed functional and anatomical damages and related mechanisms through Aβ-derived neurotoxicity by associating them with the characteristics of the olfactory system. Taken together, we show that region-specific damage of OSNs induces partial olfactory dysfunction, and this may be a feature of AD-specific olfactory dysfunction.

## Methods and materials

### Key resources table

**Table:** 

Reagent or resource	Source	Identifier
Antibodies		
Anti-Aβ-oligomer (A11)	Invitrogen	#AHB0052
Anti-6E10 (Aβ_1–16_)	Covance	#SIG-39300
Anti-D54D2 (Aβ_1–40_, Aβ_1–42_)	Cell signaling	#8243
Anti-BrdU	Thermo	#MA3-071
Anti-OMP	WAKO	#019-22291
Anti-synaptophysin	Dako	#M0776
Anti-TH	Santa Cruz	#sc-14007
Anti-Ki67	Cell signaling	#12202
Chemicals		
Fura-2	Promo Cell	PK-CA707-50034
BrdU	Sigma-Aldrich	#59-14-3
Acetophenone	Sigma-Aldrich	#42163
Allyl phenylacetate	Sigma-Aldrich	#W203904
Eugenol	Sigma-Aldrich	#E51791
Geraniol	Sigma-Aldrich	#163333
Heptanal	Sigma-Aldrich	#W254002
Heptanoic acid	Sigma-Aldrich	#75190
Lyral	Sigma-Aldrich	#95594
Mineral oil	Sigma-Aldrich	#M5904
Experimental models: organisms/strains		
5xFAD (Tg6799)	K.A. Chang’s Lab	
C57BL/6 J	KOATECH	http://www.koatech.co.kr/
Software and algorithms		
ImageJ	NIH	https://imagej.nih.gov/ij/
Fiji	NIH	https://imagej.nih.gov/ij/
Prism Software	GraphPad Software, Inc., La Jolla, USA	https://www.graphpad.com/scientific-software/prism/

### Method details

#### Animals

All experimental protocols were approved by the Institutional Animal Care and Use Committees of DGIST (DGIST-IACUC_0104). All applicable guidelines for the care and use of laboratory animals from the National Institutes of Health Guide were followed. Only male animals were used in this study. Adult mice (C57/BL6, 2-month-old) were obtained from KOATECH (Daegu, Korea). 5xFAD transgenic mice harboring the mutated human APP (695 amino acids) and human PS1 genes (Tg6799, 3–4 months old) were obtained from Prof. K.A. Chang (Gachon Medical School, Incheon, Korea). All behavioral, physiological, and histological analyses using animals in this study were blind tested.

#### Behaviors

We conducted behavioral experiments with the isolated preparation.

##### Y-maze test

The test was performed to evaluate the ability of the mice to act in a sequence and to measure short-term memory. Each branch (A, B, and C) of the maze was 40 cm long, 5 cm wide, and 10 cm high at an angle of 120°. The maze was constructed of white polyvinyl plastic. The animal was placed in the maze for 8 min, and the frequency with which the tail entered each branch was counted for each branch. The number of times the animal entered the branches (in the A, B, C sequence) was also counted and awarded 1 point (real change, actual alternation). Ability to take action to change (%) = actual change (actual alternation)/maximum change (maximum alternation) × 100 (maximum change: the total entrance number − 2).

##### Morris water maze (MWM) test

The test was conducted in a circular pool (diameter 90 cm, height 45 cm, outer height [from the ground] 61.5 cm) using the EthoVision Maze task system (Noldus Information Technology, Wageningen, the Netherlands). The time required to find the platform and the latency to escape (escape latency) was measured. The animals underwent four training trials per day (one time per quadrant) over 4 consecutive days with a 30 min interval. If the animal could not find the platform within 60 s, they were placed on the platform for 20 s. The platform was removed on the last day, the animals were placed in the water to swim for 60 s, and memory was compared by measuring swimming around the area where the platform was installed. The C57/BL6 mouse was used for verifying the background feature of the adult olfactory system. The 2-month-old mouse was considered as an adult. In our experimental condition, we compared all values from wild-type mice with age-matched.

##### Food-seeking test

The test was performed in 3-month-old 5xFAD mice (WT, *n* = 6; 5xFAD, *n* = 6) and as described previously [11]. Prior to the food-seeking tests, food restriction was applied for over 35 h to motivate animals to search for food, either hidden underneath a layer of bedding or not. Therefore, this test was used to assess latency in finding food as the buried pellet-seeking test. Mice were habituated in a clean home cage for 1 day prior to testing. A food pellet was buried 5 cm under the bedding in a middle region of the edge of the cage and a mouse was placed at the opposite edge. The time to the first bite of the food pellet was measured using an installed digital camera (maximum recording time was 10 min based on the assumption that food-restricted mice that fail to use odor cues to locate the food within a 10-min period are likely to have deficits in olfactory abilities). In data analysis and figure, we normalized the data by an average of wildtype’s time latency to evaluate effectively how time latency would be changed in a transgenic mouse.

##### Odor detection (nose poke) tests

**Fig. 1:**
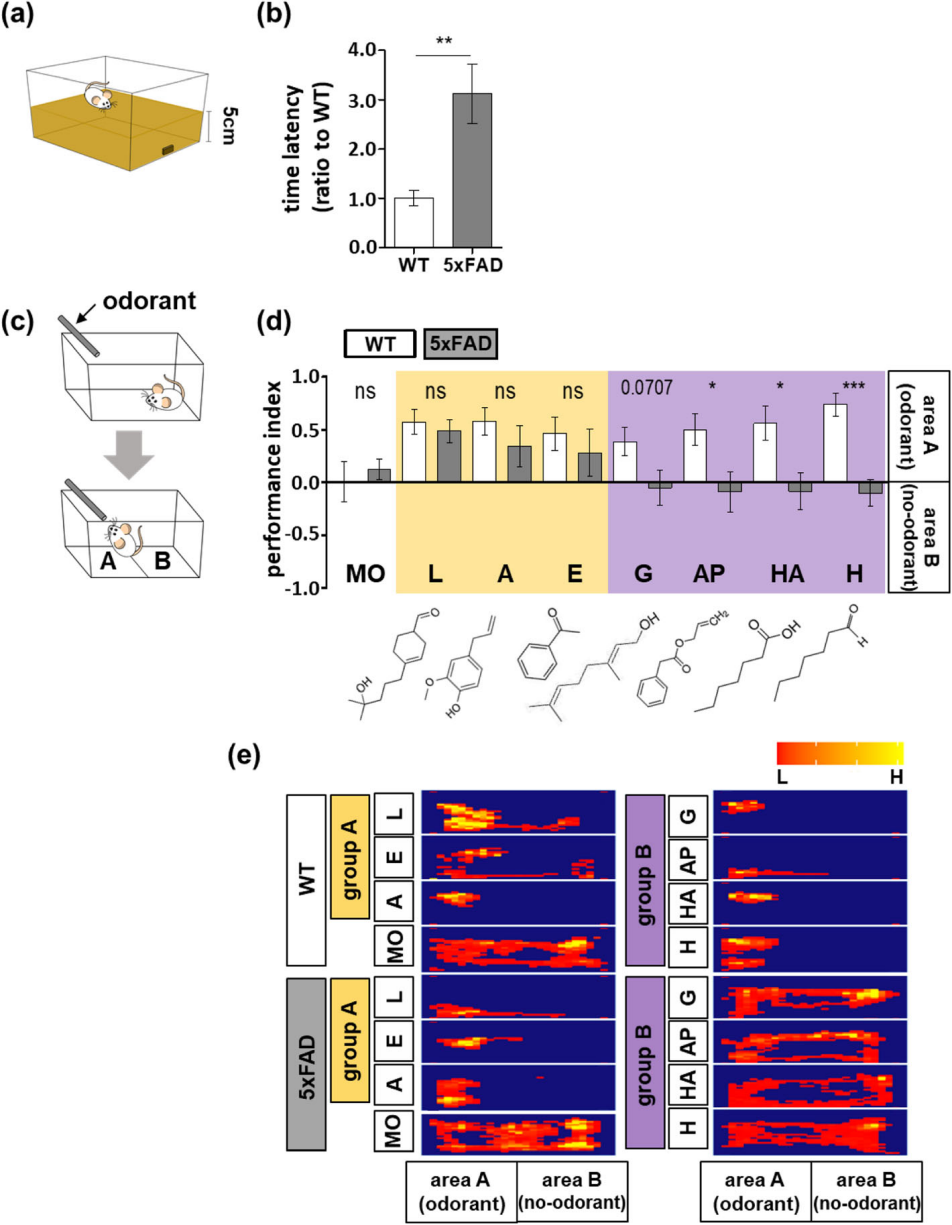
Atypical, AD-like olfactory behavior in 5xFAD transgenic mice. Experimental subjects are 3-month WT or 5xFAD mice unless otherwise noted. **a**, **b** Food seeking test (WT, *n* = 6; 5xFAD, *n* = 6). **a** Scheme illustration. **b** The latency was measured as mean ± SEM. For statistical analysis, an unpaired *t* test was performed. **c**–**e** Odor detection test was performed (WT, *n* = 6; 5xFAD, *n* = 6). **c** Scheme illustration. For PI analysis, areas **a** and **b** were defined to distinguish the location of the experimental odorant in the cage. **d** The PI value is represented as mean ± SEM. The experimental odorants refer to lyral [L], eugenol [E], acetophenone [A], geraniol [G], ally phenylacetate [AP], heptanoic acid [HA], and heptanal [H]. For statistical analysis, an unpaired *t* test was performed. **e** Representative heat map showing duration (H; high, L; low). Statistical significances are denoted as follows: ns, non-significant; *P <  0.05, **P <  0.01, ***P <  0.001). Wild-type (WT), five familial AD mutations (5xFAD), Alzheimer’s disease (AD)

The test was performed at 3 months of age for the Tg6799 and wild-type (WT) groups (*n* = 6 per group) and by modifying the odor-preference test described previously [19, 23]. Instead of filter paper, a cotton tip scented with odorant was used to allow for the nose poke of mice to more accurately direct them to the odor (Fig. 1c). The following commonly used odor preference test odorants were used and presented for 2 min: acetophenone (8.6 M), allyl phenylacetate (5.9 M), eugenol (6.5 M), geraniol (5.7 M), heptanal (7.2 M), heptanoic acid (7.1 M), and lyral (4.7 M). Mineral oil (MO) was used as a control odorant. After 5 min habituation, mice were transferred to a new cage and the tip scented with a test odorant carefully set so that the mouse could not directly reach it. Investigation times were measured for 2 min. The performance index (PI) was determined based on a previously described method [24]. PI is the percentage of time to detection of the experimental odorant minus the percentage of time in the control. A PI close to 0 indicates difficulty in detecting an odor and a PI of 100 indicates that a mouse could definitively detect an odor. The mouse behavior was recorded with a digital video camera (rate of 30 frames/s). Four points of interest (POIs) that we tracked in each frame were the nose, ears, and tail (Movie S1). In each group, we first randomly selected 180 frames and manually labeled POIs in those frames and used them to train and test a neural network model implemented in DeepLabCut [25]. Evaluation of labeling accuracy was achieved by comparing the labels acquired from the convolutional neural network on the test set with manual labels. The model was then used to evaluate all frames in each group of the 20 videos used for training. The resulting *x* and *y* coordinates corresponding to the middle position of four POIs within each frame were used to determine location. The test cage was divided into equally sized three compartments, and the duration that odorant part of mice stayed inside the third that contains a cotton tip was analyzed for odor attraction.

#### Calcium imaging

We conducted calcium imaging in the in vivo conditions with the isolated preparation of instrument.

##### Mice preparation

Imaging was performed at 3 months of age for the Tg6799, age matched WT, and C57BL6 mice (*n* = 5–6 per group). Prior to the operations and administration of a calcium indicator (Fura-dextran), mice were anesthetized with a mixture of ketamine (90 mg kg^−1^) and xylazine (10 mg kg^−1^). Calcium dye was administrated by nasal administration of a calcium indicator (using PE-90 tubes attached to a micropipette). A volume of 5–6 μl (8 μl mixture of 8%/0.2% calcium dye/Triton-X) was administered to each nostril. Six hours after the operation, mice were anesthetized again with a mixture of ketamine (90 mg kg^−1^) and xylazine (10 mg kg^−1^) and decapitated. The whole olfactory system (MOE and OB) was dissected and peeled off. The sample fixed on 4% low-melting agarose (Sigma A9414, St. Louis, MO, USA) and transferred into Ringer’s solution (140 mM NaCl, 5 mM KCl, 1 mM CaCl_2_, 1 mM MgCl_2_, 10 mM HEPES, 10 mM glucose, 1 mM Na pyruvate, pH 7.4). The perfusion rate was 2 ml/min. The sample was flattened by trimming, transferred to a tissue slice chamber (Warner instruments) with the lateral side facing up.

##### Odor stimulation

Odorants were presented as dilutions from saturated vapor in cleaned air using a custom olfactometer which was designed to provide a constant flow of air blown. The olfactometer was built by using manually controlled valves, Teflon tubing, glass syringes (10 cc), scintillation vials, flowmeter, and air pump (600 ml/min). Odorant with specific concentration identified through behavioral experiments are made with saturated vapor, and they are adding on airflow through the odorant tube and delivered directly to the olfactory system. All the odorants diluted in mineral oil (0.0001%) or mineral oil only (control) were used to generate vapor saturation in the syringes. For the different odorant, different odorant tubes set (Teflon tubing, glass syringes (10 cc), scintillation vials) were used to prevent contamination in the experiment (Fig. S2a).

##### Optical recording

A typical stimulation protocol was a 20-s duration including 2-s stimulation repeated 10 times separated by 1 min between stimulations. The bulb surface of wide-field optical signals was measured using a Nikon 10×, 0.5 NA (2.3 × 2.3 mm field of view), Nikon 16× 0.8 NA (1.4 × 1.4 mm field of view) with a 150 W Xenon arc lamp (Opti Quip), objectives with a 200 mm focal length lens for single bulb measurements. For fluctuation of calcium monitoring, we used 380/10 nm (Chroma, ET380x) excitation light and a 510/84 nm emission filter (Semrock FF01-510/84). Fluorescence emission was recorded with a NeuroCCD-SM256 camera with 2 × 2 binning between 25 and 125 Hz using NIS-Elements software (Nikon).

##### Data analysis

All target points for analysis were selected on the sagittal view of OB, aligned manually to each mouse, guided by skull features and overall outline connecting OE-OB-brain. In order to confirm the correlation with the IHC results to be followed, we analyzed the (ΔF/F_0_) change across OB to compare with the IHC analysis. Dorsal to the ventral line for our calcium analysis within OB were aligned to proceed correlation analysis with the coronal sectioned IHC analysis. The entire analysis area of OB is divided equally among 10 sections from the anterior to posterior and 180 sections from dorsal to ventral within a lateral olfactory bulb view, respectively (Fig. S2b).

Activity maps that could reflect the positional variability of glomeruli among individuals for the analysis of calcium activities are adopted. The activity map is defined by the selected target point which is automatically calculated using the LC Pro plugin for ImageJ and an outline with a distance of 200 μm from the selected target point using C57BL6 mice.

The 10 times recordings per each odorant for the Tg6799 and age-matched WT were averaged per 1 s and processed for 20 s for further analysis. The individual trials were manually inspected, and occasional trials with obvious artifacts were discarded. Then, resting-state (airflow without odorant) data (baseline fluorescence (F_0_)) was intensity normalized with averaging frames over 3 s for 10 trials before stimulus onset. Activation data with odorant stimulation was calculated with fluorescence signal difference of every time point (ΔF) which was subsequently normalized (ΔF/F_0_). Calcium measurement (ΔF/F_0_) of specific activity maps were scaled with the averaged intensity value per each time point.

To perform the correlation, a heat map analysis was also performed on only the subsets of pixels overlaying the ROIs identified in each preparation’s activity map using unfiltered activity maps. All experiments were performed and evaluated by five independent tests.

#### Tissue preparation

Animals were anesthetized by intraperitoneal injection of 65 mg/kg ketamine with 5 mg/kg xylazine. The mice were then transcardially perfused with prechilled phosphate-buffered saline (PBS, pH 7.6). Heads were removed, skinned, and post-fixed overnight in 4% paraformaldehyde in PBS at 4 °C. The mandibles were discarded, and the trimmed heads were skinned and fixed by immersion in the same fixative for 1 day at 4 °C. The heads were decalcified in Calci-Clear Rapid solution (National diagnostics, GA, USA) for 20 min at room temperature. After decalcification, the specimens were washed, dehydrated in increasing concentrations of ethanol, and transferred into xylene to clear the tissue. The specimens were infiltrated with paraffin and embedded. For cryosectioning, tissue was soaked in sucrose and embedded in Tissue-Tek OCT compound (Sakura Finetek Europe BV, Zoeterwoude, the Netherlands) after post-fixation in 4% paraformaldehyde. Frontal sections (coronal, 5 μm) were cut serially from the tip of the nose to the posterior extension of the OE and OB, and each section was preserved on Matsunami coating slide glass (Matsunami Glass Co., Tokyo, Japan).

#### Immunohistochemistry (IHC)

For IHC, the tissue was permeabilized in PBS-T (0.1% Triton X-100 in PBS) for 15 min. The endogenous peroxidase in the samples was quenched using 3% hydrogen peroxide in 10% methanol for 30 min. To retrieve antigenicity, the samples were boiled in 0.1 M citrate buffered saline (pH 6.0) for 5 min. The sections were cooled for 30 min and then washed twice in PBS (5 min each). After washing in PBS-T for 30 min, the sections were blocked for 1 h in blocking solution (4% normal donkey serum in PBS-T) and incubated with primary antibodies overnight at 4 °C. Anti-Aβ oligomer (1:100) and anti-bromodeoxyuridine (BrdU; 1:250) antibodies were used. After washing in PBS-T, the sections were incubated with a biotinylated secondary antibody for 1 h at room temperature. Sections were subsequently treated with the avidin-biotin-peroxidase complex (Vectastain Elite ABC kit) for 1 h at room temperature. The sections were developed for 5 min in a 0.05% DAB solution and counter-stained with hematoxylin. Images were captured with a Nikon digital camera (DS-Ri1) attached to a Nikon-Eclipse-90i microscope (Nikon Corp., Tokyo, Japan).

#### Immunofluorescence (IF)

For immunofluorescence, tissues were permeabilized in 0.1% PBS-T for 15 min. To retrieve antigens, the samples were boiled in 0.1 M citrate buffered saline (pH 6.0) for 5 min. The sections were cooled for 30 min and then washed twice in PBS (5 min each). After washing in PBS-T for 30 min, the sections were blocked for 1 h in blocking solution (4% normal donkey serum in PBS-T). The sections were incubated with primary antibody overnight at 4 °C. Anti-oligomer A11 (Invitrogen, CA, USA) (1:100), Anti-6E10 (Aβ_1–16_) (Covance, NJ, USA) (1:500), Anti- D54D2 (Aβ_1–40_, Aβ_1–42_) (Cell Signaling, MA, USA) (1:500), anti-synaptophysin (Agilent Dako, CA, USA) (1:250), anti-TH (Santa Cruz Biotech, TX, USA) (1:250), and anti-Ki67 (Cell Signaling, MA, USA) (1:250) antibodies were used. Alexa 488 and Cy3-conjugated secondary antibodies (Jackson Laboratory, Bar Harbor, ME, USA) were used. The sections were counter-stained and mounted using VECTASHIELD mounting medium with 4′,6-diamidino-2-phenylindole (DAPI; Vector Laboratories, CA, USA). The images were visualized and photographed by confocal fluorescence microscopy (Carl Zeiss, Thornwood, NY, USA).

#### TUNEL staining assay

For TUNEL staining, deparaffinized and rehydrated sections were washed in PBS for 5 min and treated with Proteinase K (10 μg/mL) in PBS at room temperature for 30 min. After they were washed with distilled water for 5 min, the TUNEL incubation solution (Promega, WI, USA), containing the TdT enzyme solution and label solution, was prepared following the manufacturer’s protocol. The sections were incubated in the TdT enzyme and label mixture for 1 h at 37 °C and then washed three times with PBS (5 min each). Fragmented DNA was visualized as green fluorescence inside the nuclei.

#### BrdU assay

BrdU (Sigma, St. Louis, MO) was injected and detected with an antibody recognizing BrdU. For acute labeling experiments, 100 mg/kg BrdU was injected 1, 3, and 7 day(s) before sacrifice.

#### Image processing

All images were acquired using a Nikon ECLIPSE 90i microscope and a Nikon DS-Ri 1 digital camera (Nikon Inc., Japan) and LSM700 (+ Zeiss slide scanner). Digital images were processed adjusting only brightness, contrast, and color balance. The numbers of immunoreactive cells were counted manually by two independent investigators blinded to the experimental conditions. Three slides were analyzed for each animal and observed under a microscope (× 100–400). To quantify the reciprocal intensity, the intensity per unit area was measured using Image J and the color deconvolution plug-in (http://wiki.imagej.net/Colour_Deconvolution). The target unit area of images was processed using the color deconvolution tool in Image J to separate brown from other colors. The area of brown staining was then quantified and divided by the total area to yield a percentage of staining area. Stereological analyses were conducted using Prism software (GraphPad software, USA).

#### Spatial analysis

**Fig. 2:**
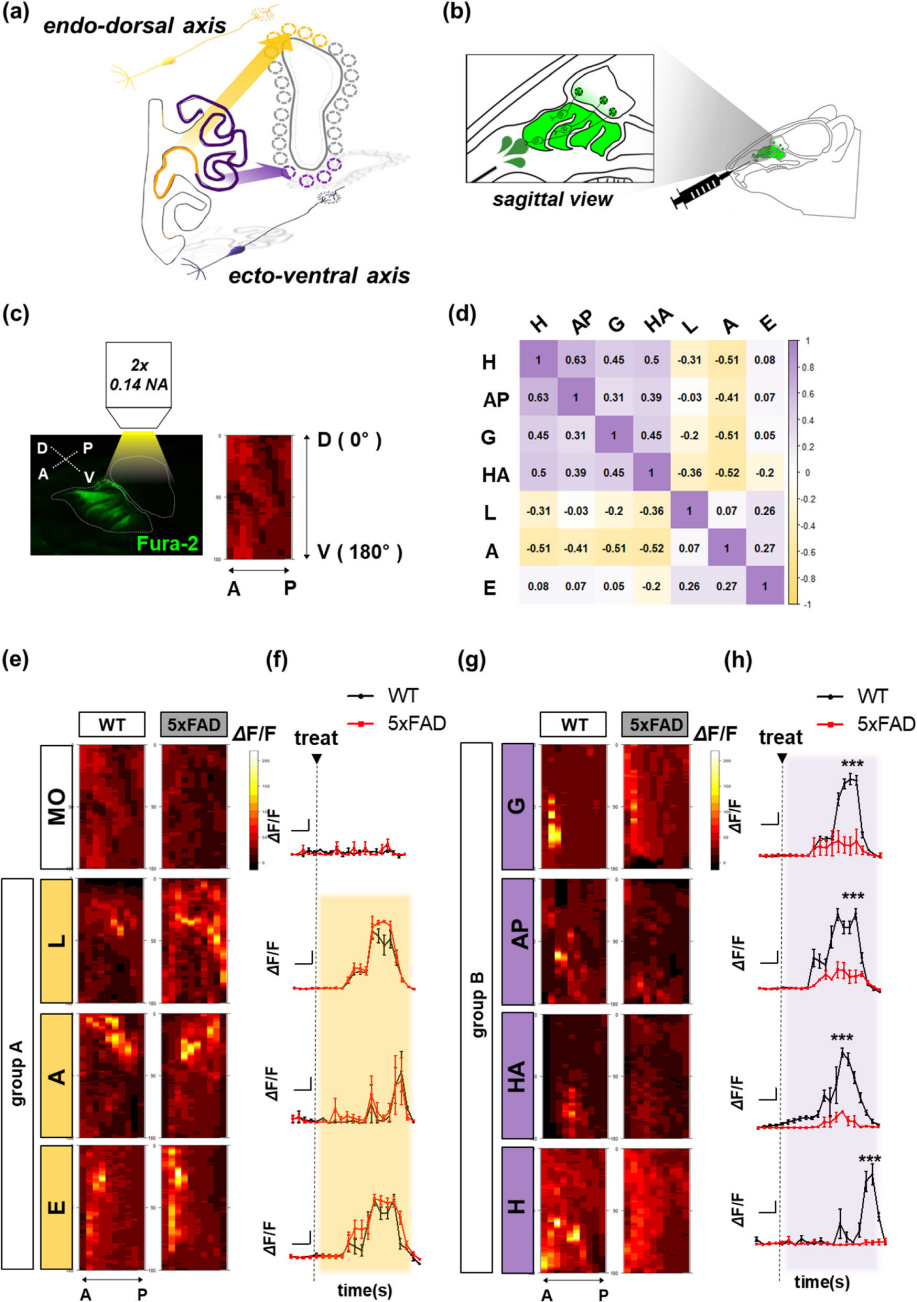
Odor-derived Ca^2+^ signal in peripheral olfactory glomeruli is divided into two groups. **a** Illustration of the topographical axis. **b** Illustration of wide-field fluorescence imaging to measure OSN-derived input signal. **c** The imaging location with high-resolution stereoscopic fluorescence image of fura-2 by OSN calcium concentration (left) and averaged activity sample map along histologic angle (D; dorsal, V; ventral, A; anterior, P; posterior) (right). **d** Heat map clustering showing the Spearman’s correlation coefficient between each active calcium signal pattern with the angle of glomeruli located. (WT, *n* = 5–6). Each odorant was divided into two groups; lyral [L], acetophenone [A], and eugenol [E] (group A), and geraniol [G], allyl phenylacetate [AP], heptanoic acid [HA], and heptanal [H] (group B). **e**, **g** The merged intensity (cumulated signal) (**f**, **h**) of olfactory synaptic activity (ΔF/F) induced by each group odorant (WT, *n* = 5–6; 5xFAD, *n* = 5–6). **e**–**f** Odor-group A. **g**–**h** Odor-group B. ΔF/F represented as mean ± SEM from five independent experiments. For statistical analysis, an unpaired *t* test was performed using Prism software (GraphPad software). Statistical significances are noted (***P <  0.001). Olfactory sensory neurons (OSNs), wild-type (WT), five familial AD mutations (5xFAD), Alzheimer’s disease (AD)

**Fig. 3:**
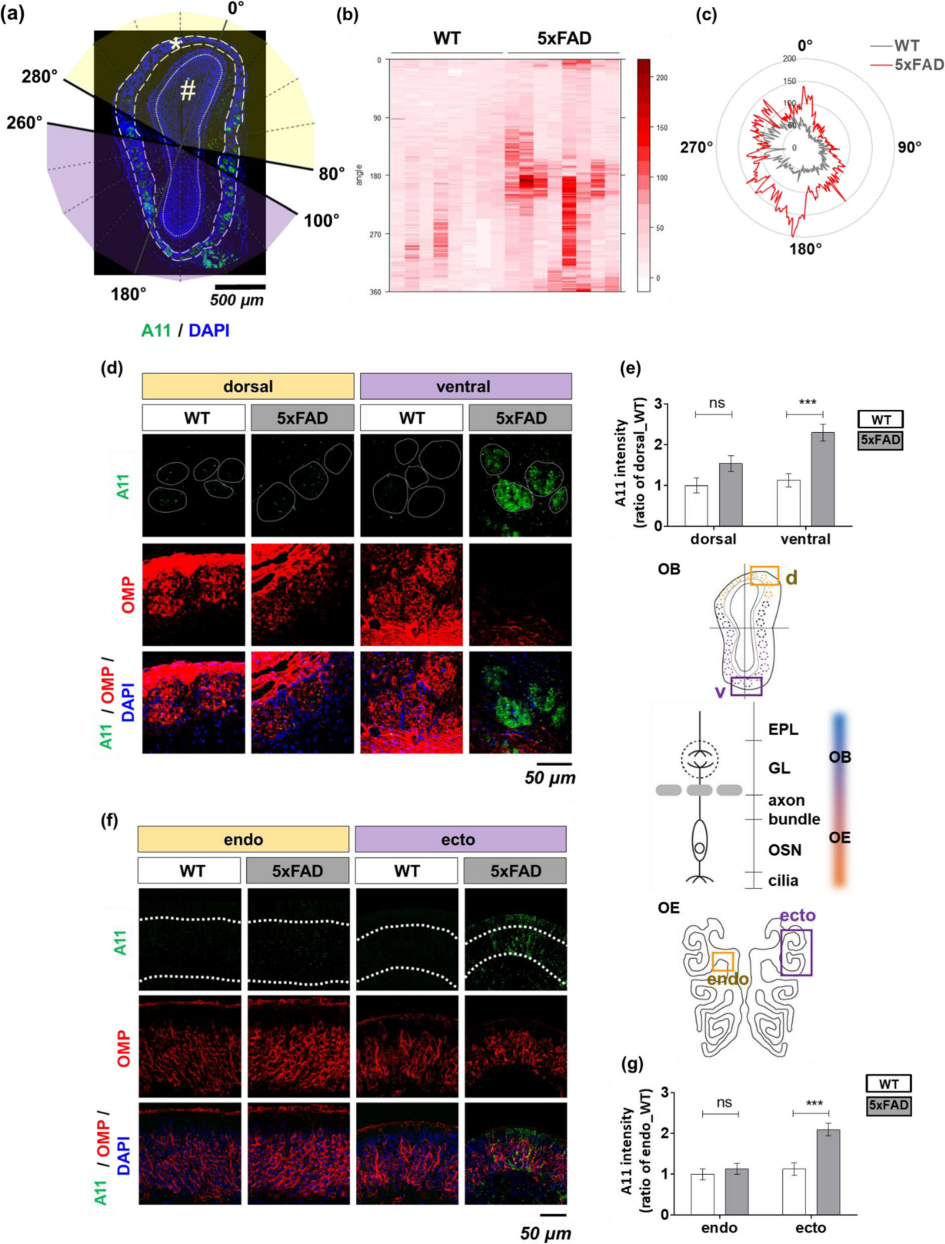
Aβ oligomer accumulation in the ecto/ventral halves of the peripheral olfactory system of 5xFAD mice. **a** Illustration of criteria to indicate the relative angular position of olfactory synapses. **b** Representative A11-positive ROI heatmap in WT (left) and 5xFAD (right) mice. **c** The radial chart represents A11-positive ROI along the angle of olfactory synapses. **d**, **e** A11 immunoreactivity was determined in the olfactory glomeruli of WT versus 5xFAD mice (WT, *n* = 7; 5xFAD, *n* = 9). **d** The local intensity of A11 (+) (green), OMP (+) (red), and DAPI (blue). **e** A11 immunoreactivity in the glomerulus (top) and topographical illustration (bottom). **f**, **g** A11 immunoreactivity was determined in the OSN layers of WT versus 5xFAD mice (WT, *n* = 7; 5xFAD, *n* = 9). **f** The local intensity of A11 (+), OMP (+), and DAPI (blue). **g** A11 immunoreactivity in OSN layers (top) and topographical illustration (bottom). All data are presented as mean ± SEM from three independent experiments. For statistical analysis, a two-way ANOVA was performed, followed by the Bonferroni post hoc test. Statistical significances are noted (ns, non-significant; ***P <  0.001). Olfactory marker protein (OMP), olfactory sensory neurons (OSNs), wild-type (WT), five familial AD mutations (5xFAD), Alzheimer’s disease (AD)

To rate olfactory synapse positions within the sectioned OB (sagittal view), we divided equally among 10 frames from the anterior to posterior within a sagittal olfactory view. The top of the glomeruli in each frame was considered as a “degree of zero” and used to set the relative angle from zero (dorsal to ventral of OB) (Fig. 2c). Spatial correlations of the calcium signal heat maps (Fig. 2d, Fig. S2e) were calculated using the function “cor” in the R software package (version 3.4.2; http://www.r-project.org/), and the correlation coefficient of each calcium signal map evoked by an odor was displayed as a heat map using the function “leverplot” in R (Fig. 2e, g). To rate olfactory synapse positions, the sectioned OB with coronal and rostral migratory stream (RMS) was used as the standard (center of the dorsal-ventral axis). The most pointed/top of the glomeruli are located along the upper RMS track and regarded as “degree of zero” (360° at the same time due to coronal view), and the ROI was measured along with glomeruli; relative angles were then based on this zero point (Fig. 3a). Spatial heat maps (Fig. 3b) representing the Aβ oligomer expression were constructed using the function “leverplot” in R. A heat map matrix represents the intensity distribution of the deconvolution of the DAB signal by the protein expression along the angle. The intensity is presented as a scale bar (0–200 *Δ*F/F) at the left side of the maps.

### Statistical analysis

**Fig. 4:**
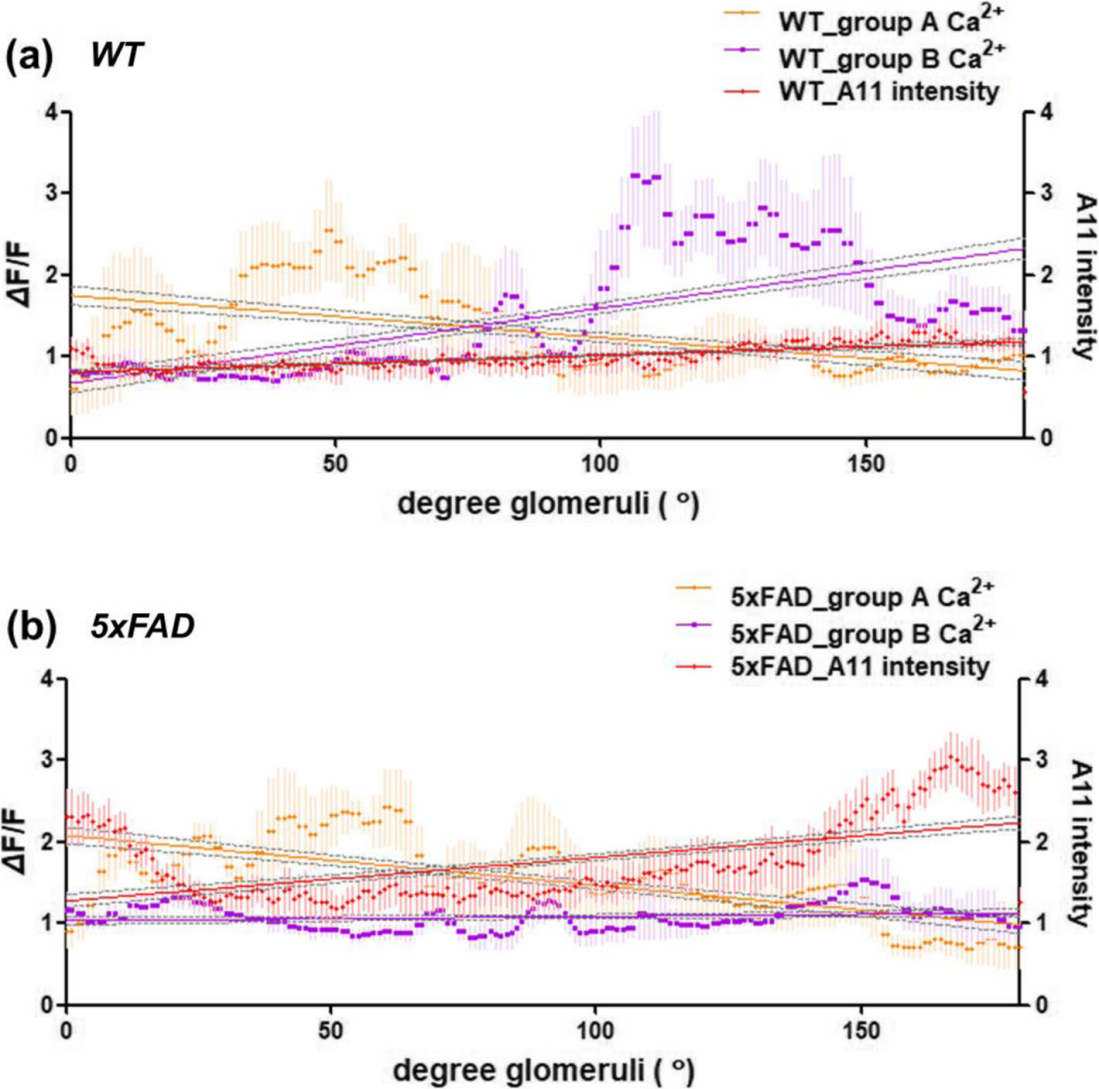
Increased A11 immunoreactivity and decreased odor Ca^2+^ activity in the ventral glomeruli of 5xFAD. **a**, **b** Scatter plots with Spearman’s correlations among the degree of glomeruli (°), A11 immunoreactivity (intensity), and odor Ca^2+^ activity in the olfactory glomeruli in WT (**a**) compared with 5xFAD (**b**) mice. Scatter diagrams displaying correlations between each variation, linear regression analysis with 95% confidence intervals was used (gray dashed line). Dot and line showing odor Ca^2+^ activity indicated in orange (group A) and purple (group B), and A11 immunoreactivity data indicated in red dots and line. Wild-type (WT), five familial AD mutations (5xFAD), Alzheimer’s disease (AD)

Statistical analyses for histological ROI evaluation were conducted using Prism software (GraphPad software, USA). Comparisons between WT and experimental mice were conducted using a *t* test. Results are presented as mean ± standard error of the mean (SEM). Differences with *P* values of ≤ 0.05 were considered to be statistically significant. Correlations were assessed with a non-parametric Spearman’s rank correlation test. Graphs (Fig. 4) show regression lines with a 95% confidence interval.

## Results

### Atypical olfactory dysfunction is observed in 5xFAD mice overexpressing Aβ

We performed food-seeking tests to examine whether olfactory dysfunction was present (Fig. 1a). Our results show that 3-month-old 5xFAD mice exhibited a significant increase in the latency time of seeking the buried food compared with wild-type (WT) mice (Fig. 1b) suggesting severe defects in olfactory function, as opposed to learning and memory dysfunction. To better understand this abnormal odor detection in the early stages of AD progression, we conducted the odor detection test in 3-month-old 5xFAD mice with several different odorants by modifying the odor preference test (Fig. 1c). We systematically compared seven different odorants for which odor detection has been associated within the main olfactory system; lyral [L], acetophenone [A], eugenol [E], geraniol [G], allyl phenylacetate [AP], heptanoic acid [HA], and heptanal [H] (0.001%). The WT mice detected all odors well and stayed near cotton tips scented with the odorants. The 5xFAD mice were able to detect lyral, acetophenone, and eugenol (odorant group A). However, geraniol, allyl phenylacetate, heptanoic acid, or heptanal (odorant group B) were not detected (Fig. 1d, e). The 5xFAD mice showed partial impairment of odor detection and not an entire loss of function. Based on this behavioral pattern, we concluded that the olfactory abnormality in 5xFAD mice has a particular uneven pattern.

In order to monitor olfactory dysfunction in the early stages of AD, we assessed cognitive and locomotor abilities that could be influenced by olfactory behavior. We performed the spontaneous alternation test and the Morris water maze task in 2-, 4-, and 6-month-old mice (Fig. S1a). We noted that total arm entries were not significantly different between WT and 5xFAD mice in the Y-maze test (Fig. S1b). The results are shown as a ratio of the results from the two cognitive tests representing the number of new arm entries in the Y-maze test and the latency time to escape the platform in the Morris water maze test. Six-month-old mice had a decreased ability to move to a new arm (Fig. S1c) and required more time to find the platform in the water maze (Fig. S1d). However, no statistically significant difference in memory impairment was found between 2- and 4-month-old mice (5xFAD as compared with WT; Fig. S1c, d). Based on these collective results, we confirmed that 5xFAD mice between the ages of 2 and 4 months display behaviors that mimic the early stages of AD progression (Fig. S1e).

### OSN-derived Ca^2+^ signals caused by odorants are decreased in the ventral glomeruli of 5xFAD mice

Because the previous results showing that olfactory dysfunction may appear in the peripheral nervous system (PNS) prior to defects in the CNS, we analyzed the sensory input signals by specific odorant stimulation in the OB [11]. It is well known that OSNs expressing a specific odorant receptor project their axons to a specific set of glomeruli in the OB. We refer to the regional projection of OSNs as the endoturbinate-dorsal glomeruli axis and ectoturbinate-ventral glomeruli axis in the peripheral olfactory system (Fig. 2a). Using these characteristics of the olfactory system, we have introduced a system that could isolate the detection function by introducing the calcium indicator (fura-2 AM) only in the OSNs (Fig. 2b). Specifically, we used a heat map model to represent the input activity from the OSN terminal of the entire OB to digitize and visualize the local information. When the OB was sagittally sectioned, the highest point of olfactory synapses (anatomically glomeruli) was noted as a “degree of zero” (dorsal side). On the contrary, the lowest point of olfactory synapses was noted as a “degree of 180” (ventral side) (Fig. 2c). To make a common odor map in the OB, we analyzed general OSN input patterns using C57 mice, a widely used inbred strain (Fig. S2c). The intensity of calcium signals by specific odorants was represented and compartmentalized with specific localization on the glomerulus layer by superimposing them over the repeated calcium image (Fig. S2d). Based on our results, the odor map grouping could be viewed from two different groups using the spatial correlation matrix: group A (lyral, acetophenone, and eugenol) or group B (geraniol, allyl phenylacetate, heptanoic acid, and heptanal) (Fig. S2e). Each odorant was classified according to a spatially correlated value. Interestingly, each of the two groups showed the same odor component in both the spatial matrix and odor detection pattern (Fig. 1e, 2d, S2e). Similar to the results obtained in C57 mice, the intensity of calcium signals by specific odorants in WT/5xFAD mice localized to the glomerulus layer (Fig. S2f). Using the localized activity map, the intensity in WT/5xFAD mice was quantified as a heat map with the spatial information (Fig. 2e, g). It was clustered into two distinct groups, the dorsally located group (group A odorants) and the ventrally located group (group B odorants) (Fig. 2d). The group A odorants presented similar patterns of calcium intensity between WT and 5xFAD mice (Fig. 2e, f, Table 1). On the other hand, group B odors resulted in a significant decrease in the intensity of calcium on the glomerulus layer in the 5xFAD mice (Fig. 2g, h, Table 1). Through both behavioral and physiological tests using specific odorants, we suggest that the spatial abnormality of OSN calcium signaling is related to the pattern of olfactory dysfunction in the 5xFAD mice.

**Table 1:** Ca^2+^ activation in the peripheral olfactory glomeruli

Unpaired ***t test***	L	A	E	G	AP	HA	H
***P*** **value (two-tailed)**	**0.2271**	**0.8298**	**0.9171**	**< 0.0001**	**< 0.0001**	**< 0.0001**	**< 0.0001**
**P value summary**	ns	ns	ns	***	***	***	***
**T**	1.287	0.2207	0.1067	8.422	9.916	12.53	9.448
**Df**	10	10	10	10	10	10	10
**Are means significantly different? (*P*** **< 0.05)**	No	No	No	Yes	Yes	Yes	Yes

Data are represented as a numerical value representing the odor-derived Ca^2+^ activity between WT and 5xFAD mice (Fig. 2f, h). The experimental odorants included lyral [L], eugenol [E], acetophenone [A], geraniol [G], ally phenylacetate [AP], heptanoic acid [HA], and heptanal [H]. Alzheimer’s disease (AD), five familial AD mutations (5xFAD). For the statistical analysis, a two-tailed unpaired *t* test was performed. Statistical significances are denoted as follows: non-significant (ns); ***P <  0.001

### OSNs accumulate Aβ oligomers with region specifically in 5xFAD mice

Next, we examined whether neurological pathology, including the olfactory defect of 5xFAD mice, is the result of Aβ oligomer formation. We examined the expression pattern of oligomeric Aβ by using A11-immunoreactivity in the OSNs. OSNs penetrate their axons into the glomerulus of the OB, which is considered as a first olfactory synapse. A11 can detect a marker of toxic soluble oligomeric Aβ [26]. By using coronal-sectioned 5xFAD olfactory tissue, A11-immunoreactivity was assessed histologically in both the OSN layer and its synapses. We also double-checked the Aβ that has different isotope using 6E10 and D54D2 (Fig. S3b). The positive signals were higher in the ventral glomerular layer (180° ± 80°), rather than other inner layers such as an external plexiform layer, mitral cell layer, and granule cell layer (Fig. S3b). We introduced a novel stereological analysis on Aβ oligomers in the 5xFAD mice. First, a two-dimensional position of olfactory glomeruli was digitized as an angle (detailed criteria provided in the “Methods and materials” section) (Fig. 3a). A heat map matrix represents the distribution of the A11-immunoreactivity in olfactory glomeruli along a specific angle (Fig. 3b, c). The A11-positive region of interest (ROI) signal was significantly enriched in 5xFAD mice, especially the ventral layer of the glomeruli (WT dorsal to 5xFAD dorsal = 1.00: 1.13, WT ventral to 5xFAD ventral = 1.54:2.30) (Fig. 3d, e). The Aβ oligomers accumulated along the topographical axis. Specifically, they were within the OSN layer of the ectoturbinate as compared to the endoturbinate in 5xFAD mice (WT endo to 5xFAD endo = 1.00: 1.13, WT ecto to 5xFAD ecto = 1.13:2.10) (Fig. 3f, g). As shown above, the uneven Aβ distribution in 5xFAD mice implies that increasing Aβ oligomers correlates with the partial dysfunction of OSNs.

### Ca^2+^ signals and Aβ accumulation are negatively correlated in the ventral glomeruli in 5xFAD mice

We conducted Spearman’s correlation analysis to examine the relationship between A11-immunoreactivity and odorant-dependent calcium activity (Fig. 4). According to angle measurements, the ROI of A11-immunoreactivity displayed a strong correlation in the ventral glomeruli (180° ± 80°) and a weak correlation in the dorsal glomeruli (0° ± 80°) of the olfactory bulb (Fig. 3c). The changes in Ca^2+^ fluorescence in the glomeruli by group A odorants (lyral, acetophenone, and eugenol) were higher in the dorsal glomeruli, and group B odorants showed higher Ca^2+^ changes in the ventral glomeruli (Fig. 4a). Interestingly, the changes in Ca^2+^ fluorescence in the glomeruli by group B odorants (geraniol, allyl phenylacetate, heptanoic acid, and heptanal) reduced in line with the strong A11-immunoreactivity in the glomeruli of 5xFAD mice (Fig. 4b), and each group B odorant showed a negative correlation with A11 intensity, which was not seen for group A (Fig. S4). The most noticeable result is that the stronger the correlation to A11 intensity, the lower the odor detection and OSN activation in 5xFAD mice (Table 2, Fig. 1d, 2h). The results indicate that decreased OSN-derived activity and odor detection in 5xFAD mice are influenced by Aβ oligomers.

**Table 2:** Correlation analysis between levels of A11 immunoreactivity and odor-induced Ca^2+^ activity at the angle-matched olfactory synapse

Parameter	Group A	L	A	E	Group B	G	AP	HA	H
**Number of XY pairs**	180	180	180	180	180	180	180	180	180
**Spearman’s rho**	**0.5951**	**0.3453**	**0.5132**	**0.4722**	**− 0.4434**	**− 0.1664**	**− 0.2983**	**− 0.4641**	**− 0.5803**
**95% confidence interval**	− 0.6849 to − 0.4897	− 0.4710 to − 0.2058	− 0.6159 to − 0.3934	− 0.5811 to − 0.3466	0.3141 to 0.5565	0.01672 to 0.3809	0.1551 to 0.4292	0.3375 to 0.5743	0.4713 to 0.6719
***P*** **value (two-tailed)**	< 0.0001	< 0.0001	< 0.0001	< 0.0001	< 0.0001	0.0251	< 0.0001	< 0.0001	< 0.0001
***P*** **value summary**	***	***	***	***	***	*	***	***	***
**Exact or approximate** ***P*** **value?**	Gaussian approximation	Gaussian approximation	Gaussian approximation	Gaussian approximation	Gaussian approximation	Gaussian approximation	Gaussian approximation	Gaussian approximation	Gaussian approximation
**Is the correlation significant? (alpha = 0.05)**	Yes	Yes	Yes	Yes	Yes	Yes	Yes	Yes	Yes

Data represent the relationship between A11 immunoreactivity and odor-induced Ca^2+^ activity at the angle-matched olfactory synapse (Fig. S4). The experimental odorants included lyral [L], eugenol [E], acetophenone [A], geraniol [G], ally phenylacetate [AP], heptanoic acid [HA], and heptanal [H]. For the statistical analysis, a non-parametric Spearman’s correlation was performed, followed by linear regression with a 95% confidence interval. Statistical significances are denoted as follows: non-significant (ns); *P < 0.05; ***P < 0.001

### Ventral periglomerular cells decrease the expression of tyrosine hydroxylase induced by input activity from ectoturbinate OSNs

**Fig. 5:**
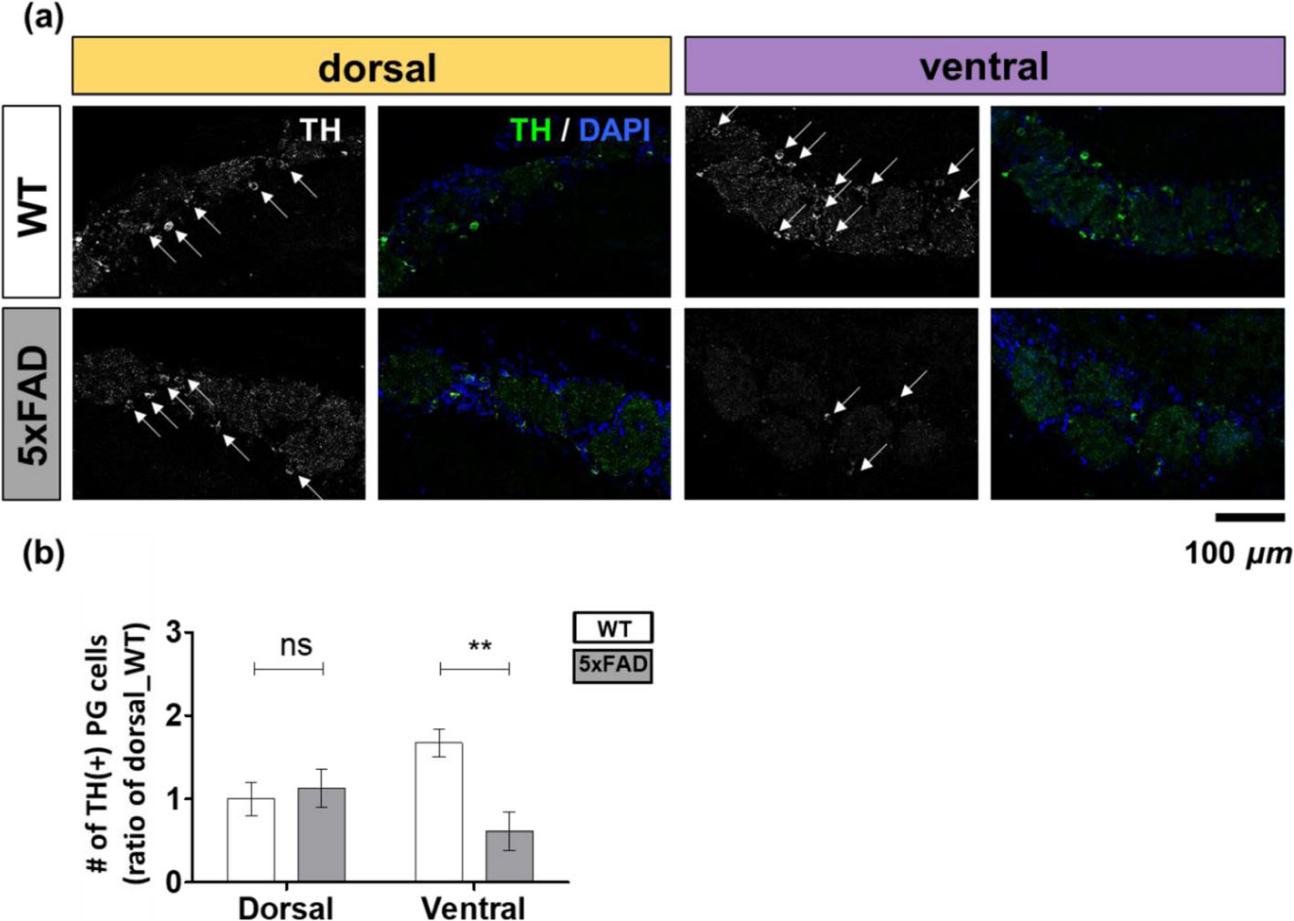
Reduction of TH expression in the ventral olfactory periglomerular cells of 5xFAD mice. **a**–**b** TH (+) (green) PG cells were identified in the olfactory glomeruli of WT versus 5xFAD mice (WT, *n* = 4; 5xFAD, *n* = 3). **a** The local intensity of TH (+) PG cells. **b** Quantitative analysis of TH (+) PG cells/5 GL cells. Data are represented as means ± SEMs from three independent experiments. For statistical analysis, a two-way ANOVA was performed, followed by the Bonferroni post hoc test. Statistical significances are denoted (ns, non-significant; **P <  0.01). Wild-type (WT), five familial AD mutations (5xFAD), Alzheimer’s disease (AD)

We measured tyrosine hydroxylase (TH) immunoreactivity in glomeruli to identify the negative effect of Aβ oligomers on the synaptic function of OSNs. This represents a marker of active OSNs. The number of periglomerular neurons expressing TH was determined as a marker of sensory input in the OSNs. The number of TH-positive neurons was significantly decreased (on average twice as low as in 5xFAD mice than in WT mice) in the ventral glomeruli (WT dorsal to 5xFAD dorsal = 1.00:1.13, WT ventral to 5xFAD ventral = 1.67: 0.61) (Fig. 5a, b). These results show that the spatially one-sided accumulation of Aβ oligomers may topographically induce partial dysfunction of the OSNs in parallel.

### Decreased maintenance of structure induced by OSN turnover disruption in the ectoturbinate olfactory epithelium of 5xFAD mice

**Fig. 6:**
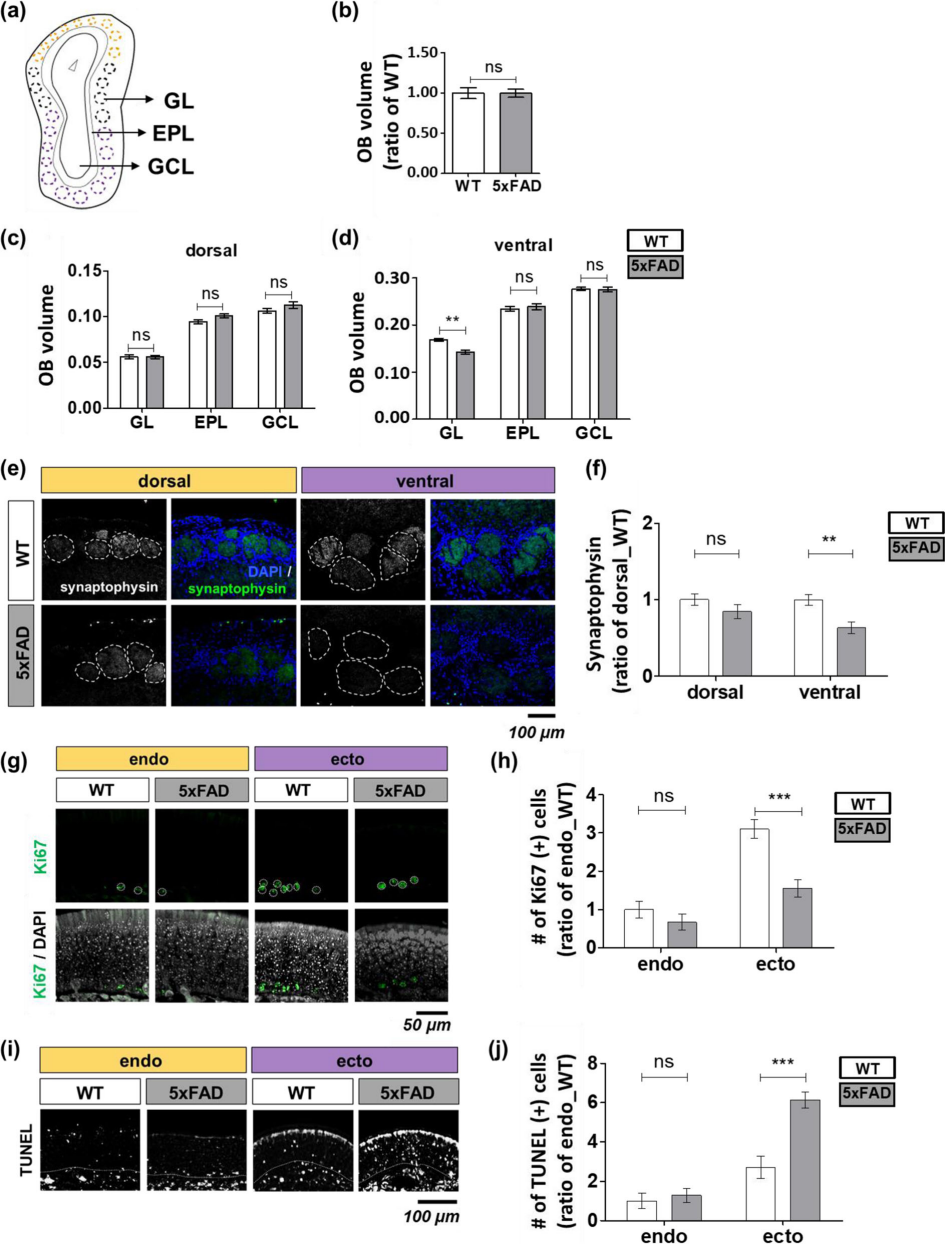
Disrupted OSN structure in the ecto/ventral region of the peripheral olfactory system. **a**–**d** Analysis of the volume of the olfactory glomerular layer (WT, *n* = 3; 5xFAD, *n* = 3). **a** Depiction of coronal sections of the olfactory bulb with laminar structure. Glomerular layer (GL), external plexiform layer (EPL), granule cell layer (GCL). **b** Total OB volume. GL, EPL, and GCL volume was measured in the dorsal olfactory synapses (**c**) and ventral olfactory synapses (**d**). **e**, **f** Synaptophysin (+) (green) glomeruli in WT versus 5xFAD mice (WT, *n* = 4; 5xFAD, *n* = 3). **e** The local immunoreactivity of synaptophysin (+) glomeruli. **f** Synaptophysin immunoreactivity. **g** Representative Ki67 (+) data in the local OSN layer (WT, *n* = 3; 5xFAD, *n* = 3). **h** Quantitative analysis of Ki67 (+) cells. **i** Representative TUNEL-positive data in local OSN layers and **j** comparative quantification (WT, *n* = 3; 5xFAD, *n* = 3). Data are represented as mean ± SEM from three independent experiments. For statistical analysis, a two-way ANOVA was performed, followed by the Bonferroni post hoc test. Statistical significances are noted (ns, non-significant; **P <  0.01, ***P <  0.001). Olfactory sensory neurons (OSNs), wild-type (WT), five familial AD mutations (5xFAD)

Neuronal synaptic dysfunction evokes interaction deficits of each neuron and leads to neural connection problems. The OB is the meeting place of the PNS and CNS, enabling a comparative study relative to an altered condition. Thus, we compared the proportion of each OB layer (Fig. 6a). The total volume of the OB did not differ between the WT and 5xFAD mice (Fig. 6b). The dorsal GL area was not significantly changed (Fig. 6c). However, the ventral glomerular layer (GL) area was decreased (WT to 5xFAD = 0.17:0.14) in 5xFAD mice (Fig. 6d). We measured synaptophysin immunoreactivity in the glomeruli to monitor the populations of presynaptic vesicles. The ROI of synaptophysin decreased in the ventral olfactory glomeruli of 5xFAD mice (WT dorsal to 5xFAD dorsal = 1.00:0.84, WT ventral to 5xFAD ventral = 0.99:0.63) (Fig. 6e, f).

Next, we examined the turnover cycle of OSNs that maintained the structure of the olfactory system. OSN proliferation and death were considered as a “turn” and “over,” respectively. The proliferating cells were evaluated by Ki67 positivity in basal cells. The number of Ki67-positive cells in the endoturbinate had a similar ratio between the WT and 5xFAD mice, but the ectoturbinate in the 5xFAD mice showed a significantly decreased number of Ki67-cells that was twice as low as in the WT mice (Fig. 6g, h). We further measured the ratio of apoptotic cell death using a TUNEL assay. The ratio of apoptotic cells was similar between the WT and 5xFAD mice. However, in the ectoturbinate, the TUNEL-positive cells increased twofold in 5xFAD mice as compared to the WT mice (Fig. 6i, j). In addition to OE, the TUNEL-positive cells in the OB were monitored in the glomerulus layer rather than the granule cell layer. Besides, the OB could not be found the region differential distribution of TUNEL-positive cells (Fig. S5). Moreover, the mature OSN marker, the olfactory marker protein (OMP) (+) OSN numbers, was decreased in 5xFAD mice, especially in the ectoturbinate (Table 3), suggesting that these changes are caused by disruption of OSN turnover.

**Table 3:** OMP (+) OSNs in 5xFAD mice compared with age-matched WT mice

	% of OMP (+) cells (mm^**2**^)	
	WT	5xFAD
Endo	65.29 ± 5.10	62.70 ± 4.04 (ns)
Ecto	71.11 ± 3.95	52.74 ± 5.25 (***)

Data are represented as percentages of OMP (+) cells in the OE (mm^2^) (means ± SDs). For statistical analysis, a two-way ANOVA was performed, followed by the Bonferroni post hoc test. *OMP* olfactory marker protein, *OSNs* olfactory sensory neurons, *AD* Alzheimer’s disease, *5xFAD* five familial AD mutations, *endo* endoturbinate, *ecto* ectoturbinate. Statistical significances are denoted as follows: non-significant (ns); ***P < 0.001

## Discussion

Previous studies have confirmed that olfactory abnormalities in AD may occur in its early stages [11, 27]. In particular, AD-related olfactory abnormalities may be specific to the peripheral olfactory system and occur prior to the increased Aβ pathology in the CNS [12, 13]. Based on the time frame we established, we confirmed the characteristics of potential AD-related olfactory problems using various odors. In order to exclude the CNS-derived effect on olfactory behavior, we examined functions that mainly involve the central nerves, including cognition, mood, and locomotion. We introduced the Y-maze test to evaluate not only cognitive tests by spontaneous arm alternation test (Fig. S1c) but also locomotor ability including motivation to move (Fig. S1b). The locomotor ability can count the number of entries when they freely move in three-arm Y-maze. According to our results from the Y-maze test, the total arm entries were not significantly different between WT and 5xFAD mice (Fig. 1b). Therefore, our Y-maze test could exclude the motor and motivational dysfunction issues influencing the observations in the food-seeking test. Additionally, these tests showed that 5xFAD mice were not affected by any abnormal behavior that can affect olfaction, such as anxiety and/or stress (Fig. S1). Although the mice were mutated so that every neuron (not specifically OSN) overexpresses Aβ, the olfactory behavior was significantly impaired before severe dysfunction of CNS behavior occurred. According to a previous study, a profound olfactory impairment has been demonstrated to precede severe memory decline and AD pathology in the brain [11]. Concerning AD progression, our experimental time point suggests that hyposmia appears at an early stage of the disease.

Each odorant has a variety of chemical features, and it is well known that the mouse may show preference when detecting odors [28, 29]. Olfactory signals are initiated in the periphery and propagate to the central olfactory system. This proceeds with three-step sequences. First, detection and identification of the odor occur via the peripheral olfactory system. Second, odor discrimination occurs, and third, odor cognition occurs via the central olfactory system. As the functional symptoms of AD are primarily related to the brain, studies of abnormal olfaction have pointed towards the dysfunction of the brain. Studies on the peripheral pathology have been overlooked, and we are the first to thoroughly illustrate the relationship between disturbances of OSNs and AD hallmarks. Clinically patients with AD with olfactory dysfunction have been reported to have an atypical dysfunctional phenotype. They are not able to sense particular odors but are not completely anosmic. They recognize the presence of odor; however, this phenomenon does not mean that they can detect all odorant information correctly. In terms of the first step in the processing of odor information, a study of OSNs of the peripheral olfactory system is required to clarify mechanisms involved in hyposmia.

Odors could be divided into at least two groups according to the behavior and physiological response in this paper. One odor group was found to activate endoturbinate—OSNs targeting the dorsal glomeruli—and the other group activated ectoturbinate—OSNs targeting the ventral glomeruli. Considering the “zone organization” theory (i.e., topographical axis), which suggests an important role for the projection from the OE to OB, it can be hypothesized that partial damage in the OE caused by AD may lead to differences in odor-specific responses. To better understand these mechanisms, we aimed to uncover the “region” involved by using various physiological analyses. It is thought that odor identity is determined by a combination of odorant receptors activated by an odorant. Activity maps are presented by each odorants’ input, and the spatial patterns of input activity are created for glomeruli. Despite the OB receiving complex odor information, humans and other animals can identify a specific odorant owing to spatial patterns across glomeruli. We attempted to quantify the relationship between the odor map by kinetics and the local information of the AD pathology of the glomerular layer in the olfactory system. The measurement applied to the degree of glomeruli suggests advanced topographical analysis in the peripheral olfactory system. This digitized measurement enables us to compare among various factors from anatomy to physiology and to confirm a correlation based on the virtual region-coordination. Our results show that odor grouping according to the odor map information (Fig. 2c) was consistent with odor grouping, in the behavioral study (Fig. 1e). Only odors from group B showed a larger difference in kinetics of 5xFAD than in the WT mice. Interestingly, the olfactory behavior corresponded with the level of calcium kinetics (Fig. 1e, Table 2). The results revealed a strong correlation between the partial behavioral defect and a pattern of low calcium kinetics. Additionally, the regional characteristics of the olfactory system in AD are associated with a topographical organization formed by the regeneration of OSNs. Further investigation on mapping OSN activity by various odorants needs to be conducted in the future to permit clearer visualization and quantification of global glomerular patterns. Although we focused only on the glomerular layer, the odor map data contains all the responses from the entire OB between WT/5xFAD mice. Thus, these methods can be easily applied to AD diagnosis by evaluating the patterns in distinct OB layers and correlating them with the glomerular patterns.

We identified the accumulation pattern of Aβ oligomers to define the relationship between abnormal behavior and AD pathology. Previous studies using transgenic mice suggested that both AD brain-derived and synthetically prepared Aβ oligomers could influence the neuronal network. The results imply that an increased threshold caused by Aβ oligomers may alter the behavioral and physiological thresholds for the detection of certain groups of odors, although more studies using various stimuli are necessary. Furthermore, Aβ could induce early synaptic toxicity [30, 31], long-term potentiation (LTP) deficits [12], and/or trigger cellular toxicity by involving tau phosphorylation and neurofibrillary tangles [32]. Moreover, we previously pointed out that Aβ oligomer toxicity in the OE may induce direct impairment of the olfactory system in early AD as OSNs express an APP-cleavage enzyme and can generate Aβ autonomously [11, 33]. Besides, oligomers are formed early in Aβ accumulation and can be harmful to the neural function and structure of olfaction in this paper. In addition to the widely known pathological effect of Aβ, recent studies have noted an interesting feature of Aβ. Aβ species are suggested as being pathological seeds and a spreading process of deteriorating proteinopathy in neurodegenerative disease [34]. Indeed, a protein pathology like α-synuclein could be associated with the toxic aggregation of proteins by being associated with endogenous Aβ expression in 5xFAD mice [35], an α-synuclein measured together with Aβ expression in the olfactory epithelium of human APP-overexpressing transgenic mice (N5 TgCRND8) [36]. Hence, Aβ present in OSNs not only can generate and accumulate misfolding protein itself but also is enough to establish systemic vulnerability in the early stage of AD. AD is a human disease, and extensive AD pathology has been shown in the human olfactory system. Not only amyloid pathology but also hyperphosphorylated tau (HP-τ) is present in the early and late stages of AD patients, and α-synuclein pathology may be seen in the OB. According to a review article by Attems et al. dealing with neuropathological findings in the OB [37], HP-τ and α-synuclein could be co-locally detected [38], and HP-τ pathology could be observed in the early stages of AD with low neuritic Braak stages before Aβ plaque occurrence. This suggests that cellular loss by HP-τ pathology may cause olfactory dysfunction in the early stages of AD [39]. They reported Aβ deposits identified from pathologically verified AD patients in the late *Braak* stage, and they reported that Aβ deposits were higher than paired helical filament tau aggregates in AD patients’ OE [40]. In accordance with the aforementioned amyloid pathology, Aβ expression can seed subsequent pathological vulnerabilities such as promoting cellular damage by HP-τ, α-synuclein, or oxidative stress [34]. Given those reports, our finding of the OSNs’ pathology can be the last stage of Aβ pathogenesis inducing the synaptic inactivity and cellular loss by HP-τ and α-synuclein pathology rather than amyloid pathology alone. Moreover, clarifying whether other proteinopathies can exhibit region-specific deposits in OSNs is a promising area for further study.

We confirmed the relationship between oligomerized Aβ proteins and odor dysfunction using 5xFAD mice. We determined the synaptic activity of our target glomeruli and confirmed the direct toxicity of Aβ. Sensory deprivation between OSNs and mitral cells induces decreasing TH expression. As dopaminergic periglomerular neurons are maturated by the excitation of glutamatergic peripheral OSNs [41], the periglomerular neurons express TH and modulate the synaptic transmission of excitatory neurons [42]. The maturated dopaminergic cells expressing TH secrete GABA in OSN axon terminals upon OSN stimulation, which contributes to presynaptic inhibition [41]. Given the physiological cascade, TH immunoreactivity has been considered as a biochemical marker of OSN activity [11, 12, 43, 44]. In the current study, a significant loss of TH-positive neurons was observed in the ventral OB of 5xFAD mice. This indicates double-confirmed data of Ca^2+^ activity representing OSN-derived input signals. Most strikingly, damaged regions were found in the ventral region, our focused region, which matched well with the diminished amounts of OMP, mature olfactory sensory receptor neurons [45]. This was also confirmed by the results of our TUNEL analyses and the reduced proliferation rate within this region. It is well known that a reduction in neurogenesis is induced by oligomerized Aβ proteins, but this is the first report showing that this event also occurs in the peripheral olfactory system.

Regarding the spatially different distributions of Aβ and partial olfactory dysfunction, we showed structural and functional deficits in the domain where OSNs regenerate greatly. The OE has topographical zones which have a distinct characteristic during their regeneration from both the external environment and intrinsic molecular mechanisms [46, 47]. Given the cellular physiology in our results, this phenomenon was intrinsically faster in the ectoturbinate than in the endoturbinate (Fig. S6). Moreover, OSNs in the dorsal domain were maintained and conserved neuronal turnover. However, the ventral domain showed higher plasticity in the normal state and deficits upon Aβ accumulation, suggesting the ventral domain has vulnerabilities such as Aβ pathogenesis. Since the OE maintains the number of OSNs by continuous proliferation and differentiation of basal cells, a reduction of this reconstitution may be a direct cause of the reduction in the number of OSNs. We can infer that the region could be the crossing point between regeneration and degeneration because APP and its cleavage enzyme secretases give rise to not only Aβ generation but also neural development and axon guidance related to the synaptic formation and neural reconstitution [48, 49]. Taken together, our result establishes that the ventral region may provide the pathogenic pool and initiate a vicious cycle of AD pathogenesis derived from Aβ, such as hyper inflammation as well as HP-τ and α-synuclein pathology.

## Limitations

The main objective was to identify hyposmia accompanying in the early phase of AD using the 5xFAD mouse. This mouse line has a limitation in reflecting the complete human symptoms of AD. Nevertheless, the experiment using 5xFAD can recapitulate Aβ expression and accumulation in the neuron system, leading causes in AD. Furthermore, the experimental design was specifically employed to capture the pathological Aβ effect on the olfactory dysfunction, one of the cardinal AD symptoms. We have chosen the mouse model by rational hypothesis (Fig. S1), but we also clarify the limitations in this selection.

We delved deeper into the pathophysiologic and anatomic effects in the peripheral olfactory sensory neurons by Aβ. The 5xFAD begins to exhibit a decline in cognition, neuronal loss, and changes in LTP/LTD in 4 to 6 months of age. Although the 5xFAD is a conventional transgenic line that has not been specifically mutated in the olfactory sensory neuron, our data showed a neuronal and functional loss in 3-month-old mice. Also, we tested that the CNS-derived effects were excluded from the olfactory function, and therefore, we hypothesized that the stage mimics the early stage of AD. However, the study design that was hypothesized the early stages of AD progression should not be overlooked when interpreting the results because the early stage of AD may be relative and not always feasible for manifestation.

To test directly the function of olfactory sensory neuron, electroolfactogram (EOG) recording is one of the most reliable approaches to analyze the physiological functions of the olfactory sensory neurons. Instead, we chose calcium imaging in the glomerulus where axons of OSNs and dendrites of central olfactory neurons form synapses. Although we could not directly show the responses of OSNs in the OE, the results from the calcium imaging in the glomerulus may reflect the responses of OSNs. Therefore, it would be considered the EOG study whether the ecto-region responses in the OE are reduced or not upon specific odorants as a further study using more scents in addition to the scents used in this study.

Seven odorants were tested in the experiment based on the preference test in the previous research. Regarding the limited numbers of odorants, it could be argued that these odorants cannot fully create categories for future therapeutic applications such as diagnosis and clinical trials. Hence, further experiments should classify odor types for AD-associated olfactory dysfunction.

The number of subjects can be a limitation of the behavioral tests for our current study, even though we consider the clear statistical significance and a small standard error of the mean. Therefore, an optimization and detailed set of conditions may be required, including an increase in the number of subjects to move on to the application as a clinical diagnosis.

## Conclusions

The present study characterized hyposmia in 5xFAD mice. We found that the olfactory abnormality in 5xFAD mice was determined by the relationship between Aβ oligomers and the regions of the nervous system that are responsible for odor detection. We have also shown, through advanced topographical analysis, that the specific patterns of Aβ oligomer accumulation can attenuate the activity in our target synapses. We confirmed a negative correlation between aggregated Aβ and both OSN-derived Ca^2+^ signals and corrupted structural stability. Moreover, these effects were identified by focusing on the spatially ecto/ventral halves formed by OSN turnover, representing a feature of the peripheral olfactory system. Thus, the collapse of the peripheral system could be the greatest feature of AD-related olfactory abnormalities.

## Supplementary Information

**Additional file 1: Figure S1.** Verification of early-stage AD phenotype in 5xFAD transgenic mice. (a) Illustration of the time course for Y-maze and Morris water maze test. (b) Basic mobility tests using Y-maze showed the total number of arm entries (WT, *n* = 23; 5xFAD, *n* = 16). (c–d) Spontaneous alternation test using the Y-maze and Morris water maze test was performed to evaluate working memory (two-month: WT, n = 23; 5xFAD, n = 16, four-month: WT, *n* = 6; 5xFAD, n = 6, six-month: WT, *n* = 10; 5xFAD, n = 10). (c) A spontaneous alternation test using Y-maze was performed and the number of entries to another arm was measured (top). Scheme illustration (bottom). (d) The Morris water maze task was performed and the ratio of escape latency (WT/5xFAD) was measured (top). Scheme illustration (bottom). (e) Illustration of experimental timepoints identified in this research based on the result interpretation. One-way ANOVA was performed for statistical analysis. All data presented as mean ± SEMs. Statistical significances are noted [ns, non-significant; ***P <  0.001]. Alzheimer’s disease (AD), wild-type (WT), five familial AD mutations (5xFAD). **Figure S2** Clustering based on spatial information of the activity maps and signal size in olfactory synapses. (a) A schematic diagram of the olfactometer. Compressed air was used as the carrier gas. Olfactometer delivered mixed air and saturated with odorant vapor in the odor applicator. The flow rates of the air and the odorant vapor were controlled by a flow meter and a syringe pump, respectively. Turning-off of the suction to the outer barrel releases odorant from the end of the applicator. (b) In order to confirm the correlation with the IHC results to be followed, we analyzed the (ΔF/F_0_) change across the whole OB. The entire analysis area of OB is divided equally among 10 sections from the anterior to posterior and 180 section from dorsal to ventral within a lateral olfactory bulb view, respectively. (c–e) The spatial information of a widely used inbred strain of mouse (C57BL/6). (c) Illustration of OSNs-derived input signal map based on calcium activity of individual odorants. (d) Clustering based on the spatial correlation between each calcium activity pattern with angle glomeruli located in Fig. 2. (e) Each odorant was divided into two groups; lyral [L], acetophenone [A], and eugenol [E] (group A); geraniol [G], allyl phenylacetate [AP], heptanoic acid [HA], and heptanal [H] (group B). (f) Representative spatial information from WT and 5xFAD mice. Olfactory sensory neurons (OSNs), wild-type (WT), five familial AD mutations (5xFAD). **Figure S3** The immunoreactivity of Aβ isoforms in the coronal section of the olfactory bulb. (a) Illustration of criteria to indicate the relative angular position of olfactory synapses. (b) The immunoreactivity of Aβ isoforms in the coronal section of the olfactory bulb (green); (Top) anti-A11 (oligomeric), (Middle) anti-6E10 (Aβ1–16), (Bottom) anti-D54D2 (Aβ1–40, Aβ1–42), and DAPI (blue). Asterisk (*): glomerular layer, Hash (#): granule cell layer. Scale bar: 500 μm. Wild-type (WT), five familial AD mutations (5xFAD). **Figure S4** The angle-matched spatial correlation between A11 level in 5xFAD and calcium activity in WT. Scatter diagrams displaying correlations between each variable and linear regression analysis with 95% confidence intervals were used (gray dashed line). (a) Correlation with lyral, acetophenone, and eugenol. (b) Correlation with geraniol, allyl phenylacetate, heptanoic acid, and heptanal. Wild-type (WT), five familial AD mutations (5xFAD), Alzheimer’s disease (AD). **Figure S5** Cell death of the OB. The TUNEL-positive signal in the coronal section of olfactory bulb (green). (Top) Domain section image, (bottom) magnified cropped image from upper domain section image, and DAPI (blue). Asterisk (*): glomerular layer, Hash (#): granule cell layer, circle (O): TUNEL positive cell. Scale bar: 100 μm. Wild-type (WT), five familial AD mutations (5xFAD). **Figure S6** Intrinsic turnover characteristics of OSNs. All experimental subjects in this figure were two-month C57BL/6 mice, a widely used inbred strain. (a–c) Analysis of OSNs proliferation and differentiation (“turn”). (a) Representative IHC data quantifying BrdU (+) cells by time after BrdU intraperitoneal (IP) injection between turbinate ecto and endo. (b) Comparative quantification one day after injection between ecto and endoturbinate. (c) Differentiation ratio through quantification by migration degree by time after injection between the ecto and endoturbinate. (d–e) Analysis of the death of OSNs (“over”). (d) TUNEL (+) cells indicated as IF data in each turbinate and (e) comparative quantification. Data are represented as mean ± SEM from three independent experiments. For statistical analysis, a two-tailed unpaired t-test was performed using Prism software (GraphPad software, USA). Statistical significances are noted (*P <  0.05, ***P <  0.001). Olfactory sensory neurons (OSNs), wild-type (WT), five familial AD mutations (5xFAD), Alzheimer’s disease (AD).

**Additional file 2: Movie S1** Representative clip of trained moving mouse for analysis of odor detection test using DeepLabCut. Four points of interest (POIs) that we tracked in each frame were the nose (blue), ears (light blue and yellow), and tail (red). Randomly selected 180 frames and manually labeled POIs in those frames, and used them to train and test a neural network model implemented in DeepLabCut. Evaluation of labeling accuracy was achieved by comparing the labels acquired from the convolutional neural network on the test set with manual labels. The model was then used to evaluate all frames in each group of the 20 videos used for training. The resulting x and y coordinates corresponding to the middle position of four POIs within each frame were used to determine location.

## Data Availability

All data generated or analyzed during this study are included in this published article and its supplementary information files. The datasets used and/or analyzed during the current study are available from the corresponding author on reasonable request.

## Abbreviations

AD: Alzheimer’s disease
OSNs: Olfactory sensory neurons
FAD: Familial forms of AD
APP: Amyloid precursor protein
PS1: Presenilin 1
5xFAD mouse: Human APP and PS1 transgenic mouse with a total of five AD-linked mutations
Aβ: Amyloid-β
OB: Olfactory bulb
OE: Olfactory epithelium
CNS: Central nervous system
PNS: Peripheral nervous system
WT: Wild-type
L: Lyral
A: Acetophenone
E: Eugenol
G: Geraniol
AP: Allyl phenylacetate
HA: Heptanoic acid
H: Heptanal
C57 mouse, C57BL6: C57 black mouse
ROI: Region of interest
TH: Tyrosine hydroxylase
GL: Glomerular layer
TUNEL: Terminal deoxynucleotidyl transferase dUTP nick end labeling
OMP: Olfactory marker protein
LTP: Long-term potentiation
D: Dorsal
V: Ventral
A: Anterior
P: Posterior
IP: Intraperitoneal
POI: Point of interests
SEM: Standard error of the mean
SD: Standard deviation
IHC: Immunohistochemistry
IF: Immunofluorescence
