# Supplementary material for: Region-specific amyloid-β accumulation in the olfactory system influences olfactory sensory neuronal dysfunction in 5xFAD mice
Source: Alzheimers Res Ther. 2021 Jan 4;13:4. doi: 10.1186/s13195-020-00730-2 (PMC7784287; doi:10.1186/s13195-020-00730-2)
Supplement: Supplementary file 1 — Additional file 1: Figure S1. Verification of early-stage AD phenotype in 5xFAD transgenic mice. (a) Illustration of the time course for Y-maze and Morris water maze test. (b) Basic mobility tests using Y-maze showed the total number of arm entries (WT, n = 23; 5xFAD, n = 16). (c–d) Spontaneous alternation test using the Y-maze and Morris water maze test was performed to evaluate working memory (two-month: WT, n = 23; 5xFAD, n = 16, four-month: WT, n = 6; 5xFAD, n = 6, six-month: WT, n = 10; 5xFAD, n = 10). (c) A spontaneous alternation test using Y-maze was performed and the number of entries to another arm was measured (top). Scheme illustration (bottom). (d) The Morris water maze task was performed and the ratio of escape latency (WT/5xFAD) was measured (top). Scheme illustration (bottom). (e) Illustration of experimental timepoints identified in this research based on the result interpretation. One-way ANOVA was performed for statistical analysis. All data presented as mean ± SEMs. Statistical significances are noted [ns, non-significant; ***P < 0.001]. Alzheimer’s disease (AD), wild-type (WT), five familial AD mutations (5xFAD). Figure S2 Clustering based on spatial information of the activity maps and signal size in olfactory synapses. (a) A schematic diagram of the olfactometer. Compressed air was used as the carrier gas. Olfactometer delivered mixed air and saturated with odorant vapor in the odor applicator. The flow rates of the air and the odorant vapor were controlled by a flow meter and a syringe pump, respectively. Turning-off of the suction to the outer barrel releases odorant from the end of the applicator. (b) In order to confirm the correlation with the IHC results to be followed, we analyzed the (ΔF/F0) change across the whole OB. The entire analysis area of OB is divided equally among 10 sections from the anterior to posterior and 180 section from dorsal to ventral within a lateral olfactory bulb view, respectively. (c–e) The spatial information [file 13195_2020_730_MOESM1_ESM.pdf]

Manuscript Format: Alzheimer's Research & Therapy (Supplementary materials)

***** titles and legends are included in the main text *****

Title

Region specific amyloid-β accumulation in the olfactory system influences olfactory sensory neuronal dysfunction in 5xFAD mice

Authors

Gowoon Son[1,4]†, Seung-Jun Yoo[1, 2,#]†, Shinwoo Kang[5], Ameer Rasheed[1], Da Hae Jung[1], Hyunjun Park[5], Bongki Cho[1,2], Harry W.M. Steinbusch[4], Keun-A Chang[5], Yoo-Hun Suh[5], Cheil Moon[1, 2, 3*]

Affiliations

[1]Department of Brain & Cognitive Sciences, Graduate School, Daegu Gyeungbuk Institute of Science and Technology (DGIST), Daegu, Republic of Korea.
[2]Convergence Research Advanced Centre for Olfaction, Daegu Gyeungbuk Institute of Science and Technology (DGIST), Daegu, Republic of Korea.
[3]Korea Brain Research Institute, Daegu, Republic of Korea.
[4]School for Mental Health and Neuroscience, Maastricht University, Maastricht, the Netherlands.
[5]Department of Pharmacology, School of Medicine, Gachon Medical School, Incheon, Republic of Korea.

† These two authors contributed equally to this work

# Present address for S Yoo: Max Planck Research Unit for Neurogenetics, Frankfurt, Germany

*Corresponding author: Cheil Moon, Ph.D. E-mail: cmoon@dgist.ac.kr

Figure S1.

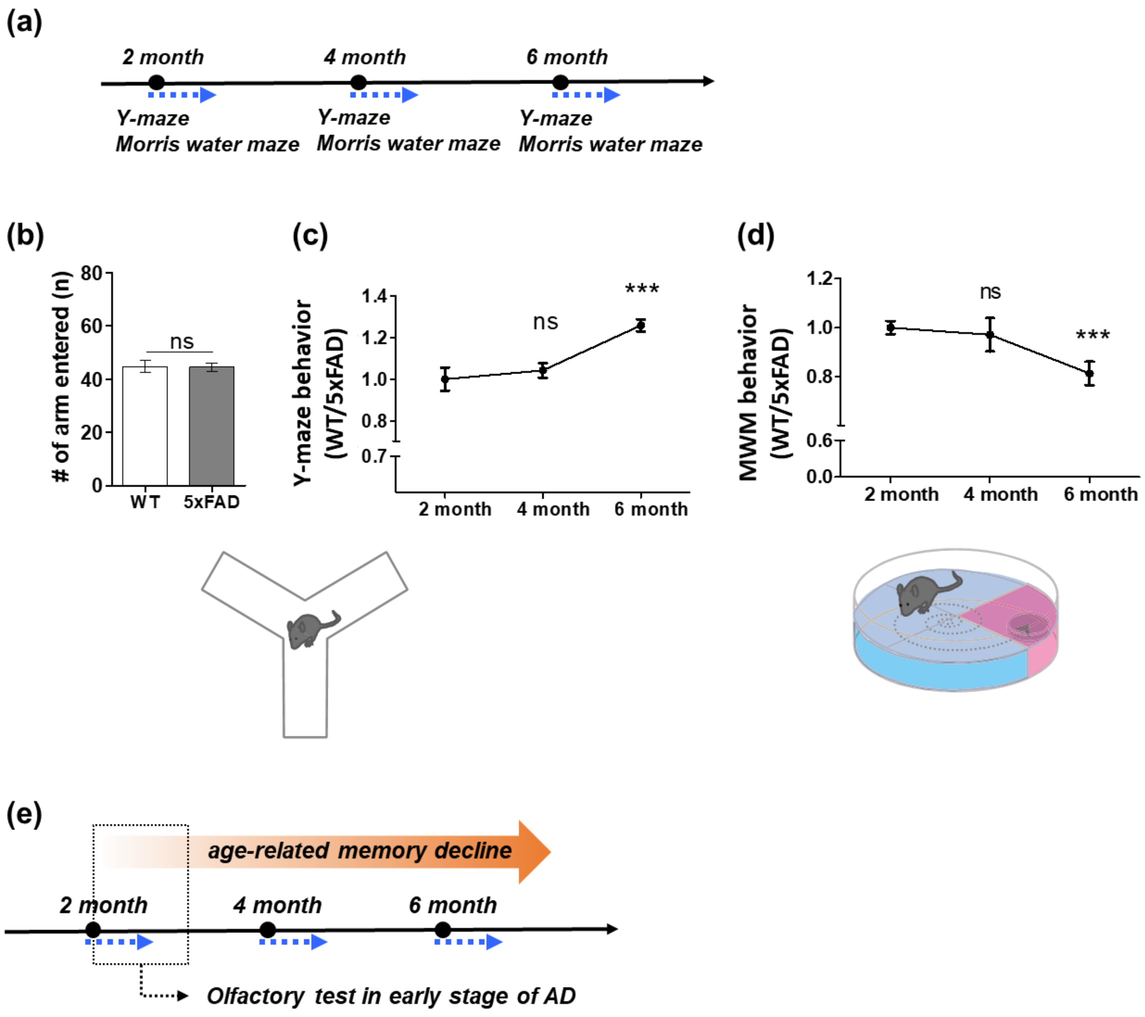

(a)
2 month
4 month
6 month
Y-maze
Morris water maze
Y-maze
Morris water maze
Y-maze
Morris water maze
(b)
# of arm entered (n)
80
60
40
20
0
ns
WT
5xFAD
(c)
Y-maze behavior
(WT/5xFAD)
1.4
1.2
1.0
0.8
0.7
ns
***
2 month
4 month
6 month
(d)
MWM behavior
(WT/5xFAD)
1.2
1.0
0.8
0.6
0.0
ns
***
2 month
4 month
6 month
(e)
age-related memory decline
2 month
4 month
6 month
Olfactory test in early stage of AD

Figure S2.

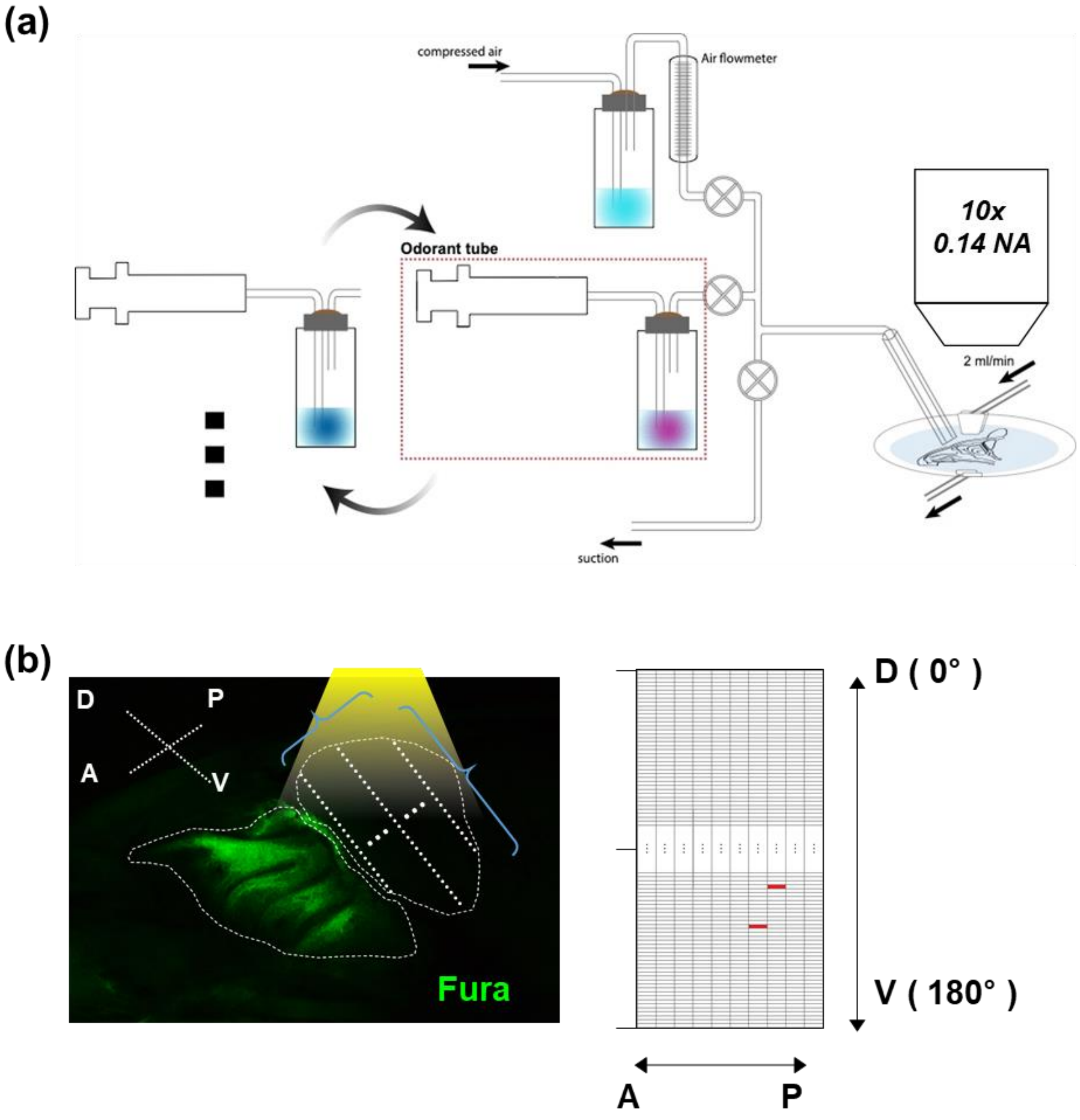
(a)
compressed air
Air flowmeter
10x
0.14 NA
Odorant tube
2 ml/min
suction
(b)
D
P
A
V
Fura
D (0°)
V (180°)
A
P

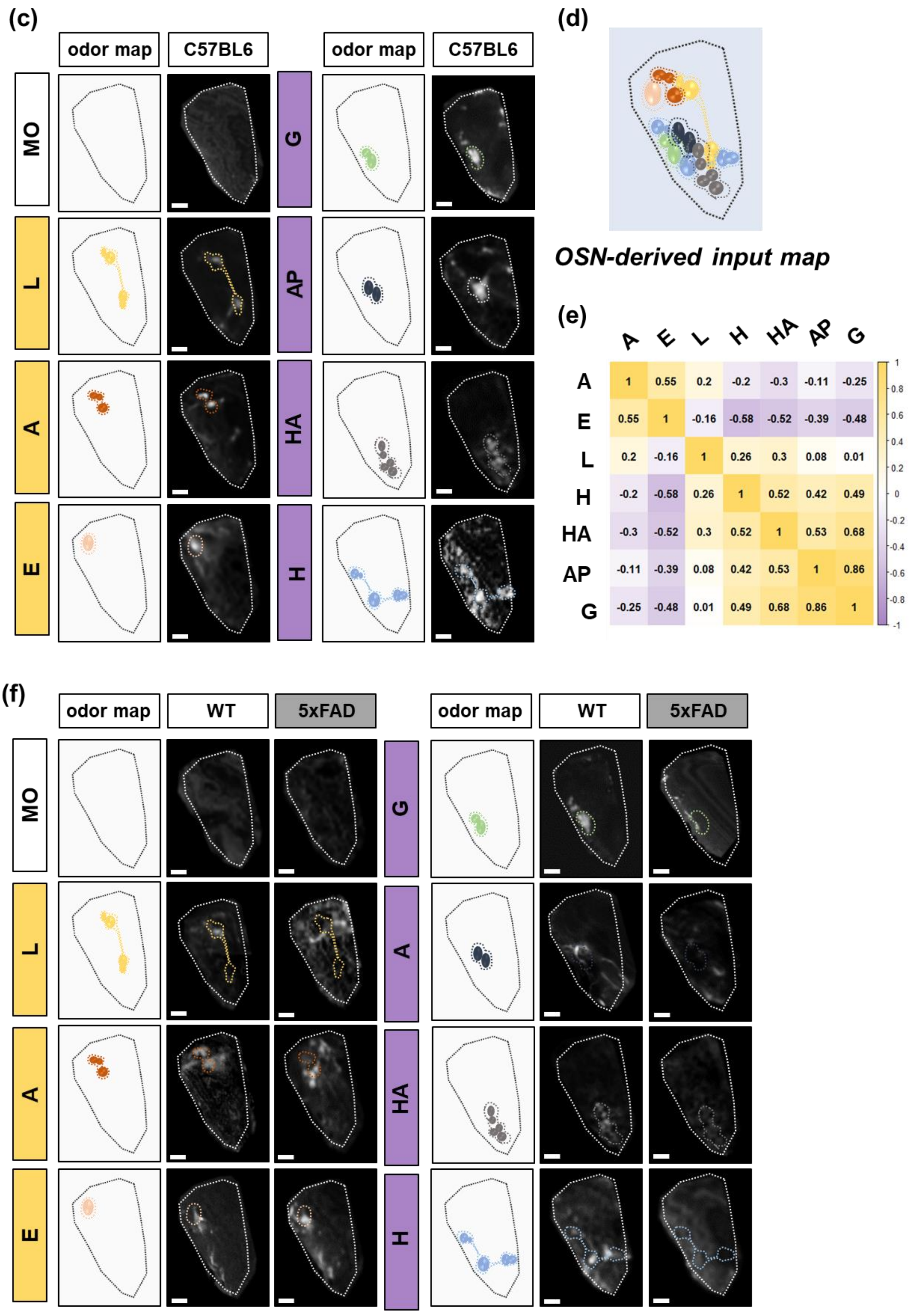
(c)
odor map
C57BL6
odor map
C57BL6
MO
L
A
E
G
AP
HA
H
(d)
OSN-derived input map
(e)
A E L H HA AP G
A 1 0.55 0.2 -0.2 -0.3 -0.11 -0.25
E 0.55 1 -0.16 -0.58 -0.52 -0.39 -0.48
L 0.2 -0.16 1 0.26 0.3 0.08 0.01
H -0.2 -0.58 0.26 1 0.52 0.42 0.49
HA -0.3 -0.52 0.3 0.52 1 0.53 0.68
AP -0.11 -0.39 0.08 0.42 0.53 1 0.86
G -0.25 -0.48 0.01 0.49 0.68 0.86 1
1 0.8 0.6 0.4 0.2 0 -0.2 -0.4 -0.6 -0.8 -1
(f)
odor map
WT
5xFAD
odor map
WT
5xFAD
MO
L
A
E
G
A
HA
H

Figure S3.

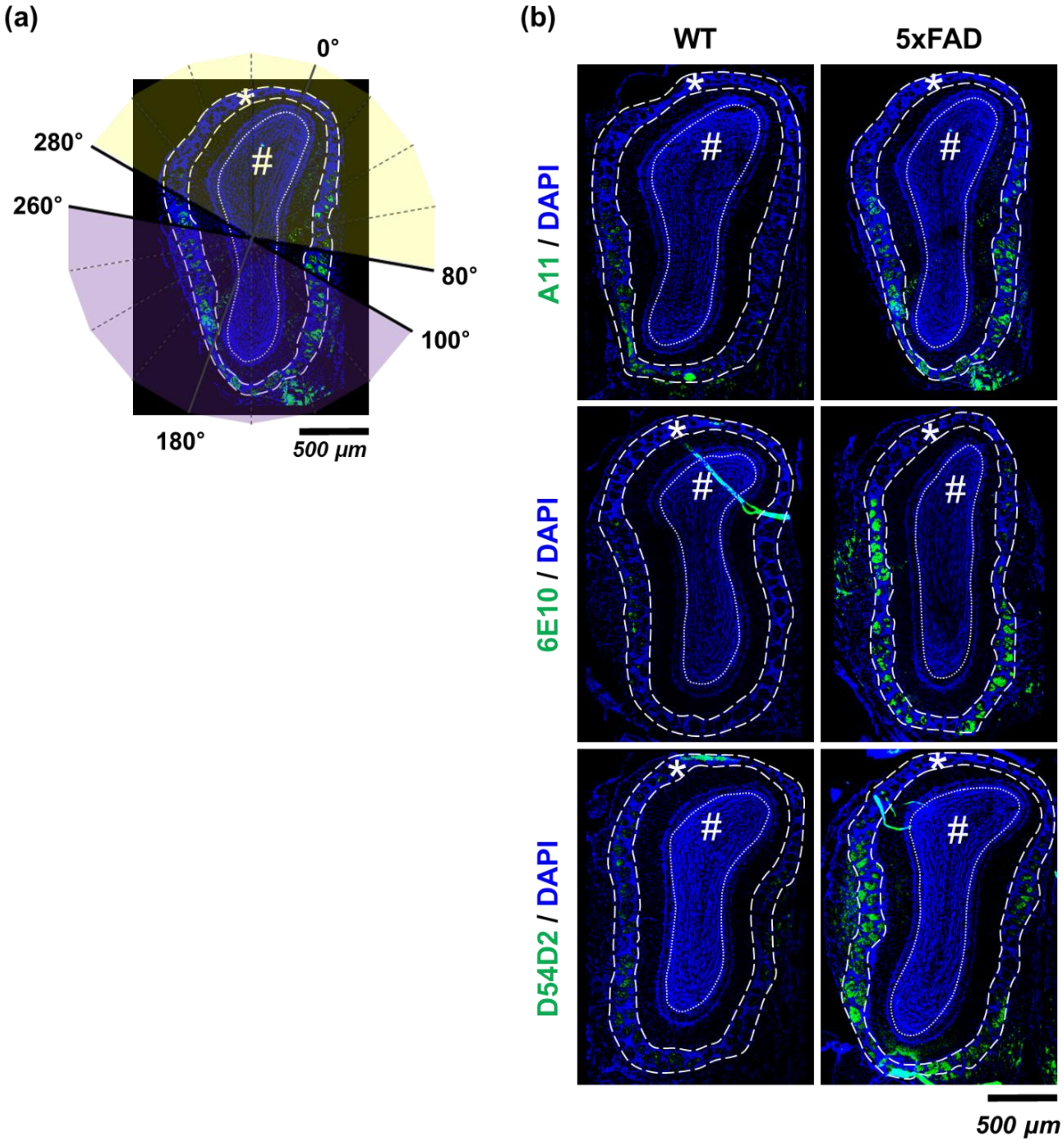
(a)
0°
280°
260°
80°
100°
180°
500 µm
*
#
(b)
WT
5xFAD
A11 / DAPI
6E10 / DAPI
D54D2 / DAPI
500 µm

Figure S4.

(a)

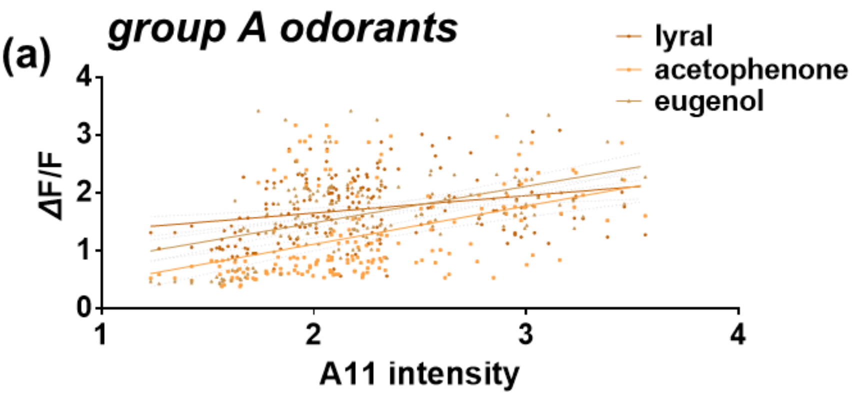
group A odorants
lyral
acetophenone
eugenol
ΔF/F
0
1
2
3
4
1
2
3
4
A11 intensity

(b)

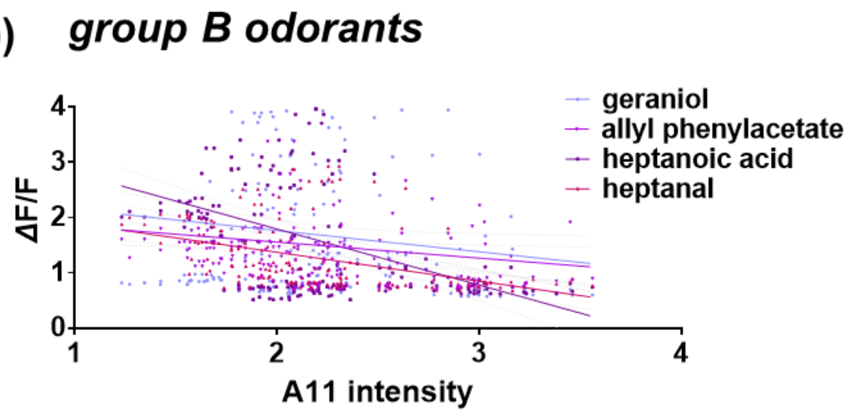
group B odorants
geraniol
allyl phenylacetate
heptanoic acid
heptanal
ΔF/F
0
1
2
3
4
1
2
3
4
A11 intensity

Figure S5.

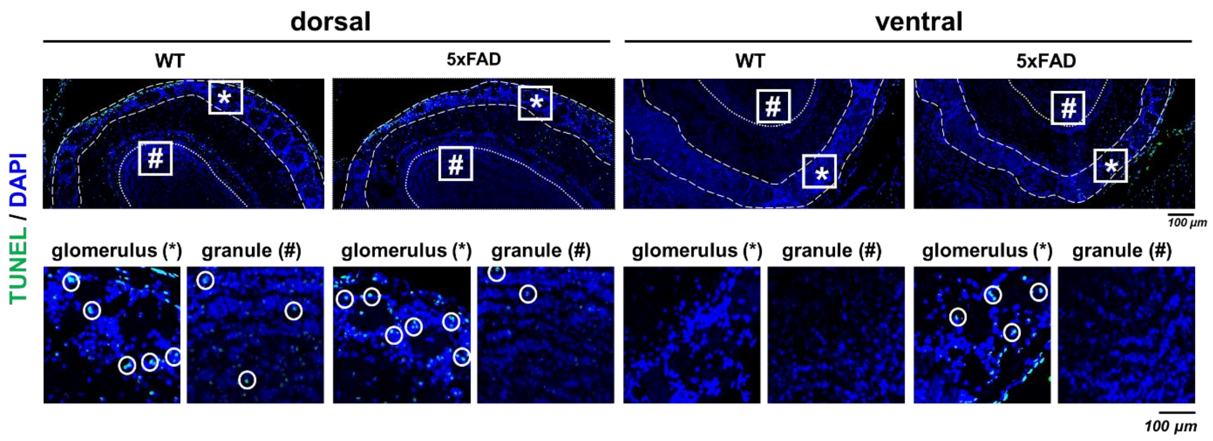
dorsal
ventral
WT
5xFAD
WT
5xFAD
TUNEL / DAPI
100 µm
glomerulus (*)
granule (#)
glomerulus (*)
granule (#)
glomerulus (*)
granule (#)
glomerulus (*)
granule (#)
100 µm

Figure S6.

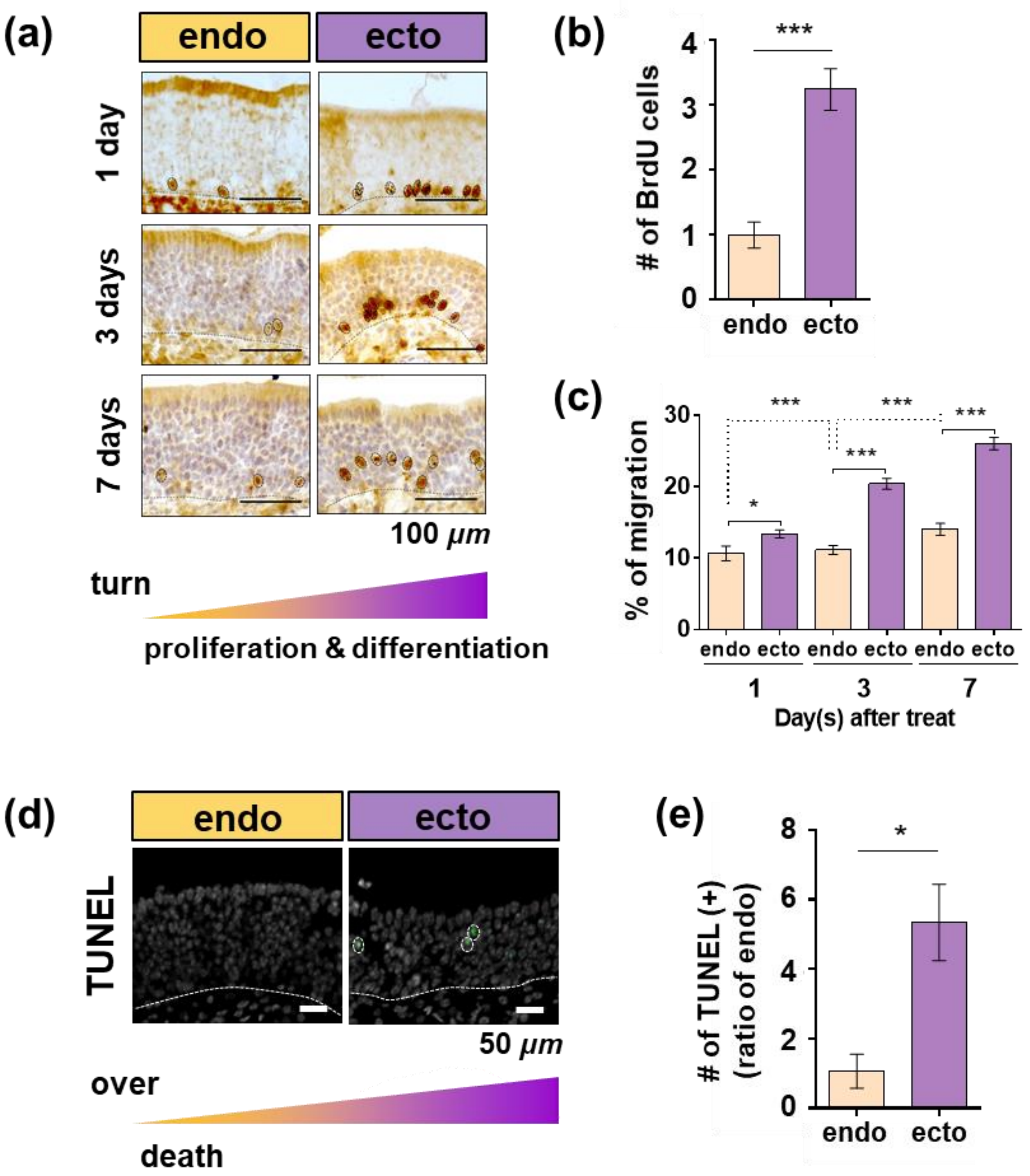
(a)
endo
ecto
1 day
3 days
7 days
100 μm
turn
proliferation & differentiation
(b)
***
# of BrdU cells
4
3
2
1
0
endo
ecto
(c)
***
***
***
***
*
% of migration
30
20
10
0
endo ecto endo ecto endo ecto
1
3
7
Day(s) after treat
(d)
endo
ecto
TUNEL
50 μm
over
death
(e)
*
# of TUNEL (+)
(ratio of endo)
8
6
4
2
0
endo
ecto
